# iDCF: Interpretable deconvolution of cell fractions via biologically-informed deep learning using scRNA-seq data

**DOI:** 10.1371/journal.pcbi.1014727

**Published:** 2026-08-27

**Authors:** Hongjiang Guo, Tingfang Wu, Wenzheng Wang, Yelu Jiang, Geng Li, Liangpeng Nie, Yunhua Jia, Lijun Quan, Moli Huang, Qiang Lyu

**Affiliations:** 1 School of Computer Science and Technology, Soochow University, Suzhou, Jiangsu, China; 2 Biomedical Basic Research Center (BBRC) of Jiangsu, Suzhou, Jiangsu, China; 3 Jiangsu Key Laboratory of Drug Discovery and Translational Research for Brain Diseases, Soochow University, Suzhou, Jiangsu, China; 4 MOE Key Laboratory of Geriatric Diseases and Immunology, School of Basic Medical Sciences, Suzhou Medical College of Soochow University, Soochow University, Suzhou, Jiangsu, China; Burnet Institute, AUSTRALIA

## Abstract

Precise resolution of cellular heterogeneity within complex tissues is fundamental to deciphering disease etiologies from bulk transcriptomic profiles. While computational deconvolution offers a scalable alternative, current deep learning methods predominantly operate as “black boxes,” neglecting the structural constraints of biological laws. This reliance on purely data-driven feature extraction often yields biologically incoherent predictions and limited mechanistic interpretability. iDCF (**I**nterpretable **D**econvolution of **C**ell **F**ractions) is a novel framework that enforces biological topology onto deep neural networks. The iDCF architecture employs a dual-stream design, synergizing a standard deep network with a knowledge-based sparse neural network (KSNN) explicitly masked by pathway definitions and protein-protein interaction (PPI) networks. In comprehensive benchmarks, iDCF achieves top-tier performance, consistently ranking among state-of-the-art methods in accuracy and robustness. iDCF integrates the SHapley Additive exPlanations (SHAP) framework, bridging the gap between computational inference and biological intuition. The model’s decision logic is governed by established biological mechanisms rather than spurious statistical correlations, validating its reliability. Validations across clinical contexts, including Alzheimer’s disease, ovarian cancer, and diabetes, demonstrate iDCF’s ability to recover disease-relevant cellular dynamics. iDCF offers a high-performance, interpretable, and biologically grounded tool for deconvolving cell-type proportions, facilitating deeper insights into tissue heterogeneity in health and disease.

## Author summary

Understanding the cellular composition of tissues is essential for unraveling disease mechanisms, yet bulk RNA sequencing only captures averaged signals from mixed cell populations. Computational deconvolution can estimate cell-type proportions from bulk data, but existing deep learning methods largely function as “black boxes,” ignoring the biological structure that governs gene expression. Here, we introduce iDCF, a deep learning framework that explicitly embeds biological prior knowledge, including signaling pathways and protein-protein interaction networks, into its neural network architecture. By constraining the model’s connectivity to biologically validated interactions, iDCF ensures that its predictions are driven by genuine biological signals rather than spurious statistical correlations. Through integration of the SHAP interpretability framework, iDCF further provides transparent, gene-level explanations for each prediction, enabling researchers to verify whether model decisions align with known biology. Extensive benchmarks across peripheral blood, brain tissue from Alzheimer’s disease patients, ovarian tumors, and pancreatic islets demonstrate that iDCF achieves superior accuracy while maintaining mechanistic transparency. We believe iDCF offers a reliable and interpretable tool for dissecting cellular heterogeneity, facilitating deeper biological insights from the vast archives of bulk transcriptomic data.

## Introduction

Understanding the precise cellular composition of tissues is a cornerstone of modern biomedicine, critical for deciphering the mechanisms governing human development, immune regulation, and disease progression [1]. In complex pathological states, such as the tumor microenvironment (TME) or neurodegenerative lesions, cellular heterogeneity often dictates clinical outcomes and therapeutic responses [2]. While bulk RNA sequencing (bulk RNA-seq) remains the workhorse for profiling gene expression in large-scale cohorts, it fundamentally measures the averaged transcriptional output of a mixed cell population. This “averaging effect” obscures cell-type-specific signals, making it difficult to distinguish between changes in gene regulation and shifts in cellular abundance [3]. Conversely, single-cell RNA sequencing (scRNA-seq) has revolutionized the field by capturing transcriptomic landscapes at cellular resolution [4]. However, the prohibitively high costs, technical noise (e.g., dropout events), and logistical challenges associated with fresh tissue handling restrict the application of scRNA-seq in large-scale epidemiological studies and routine clinical diagnostics [5]. Consequently, computational cell deconvolution has emerged not merely as a cost-saving measure, but as an indispensable analytical lens for unlocking cellular resolution from the vast archives of legacy clinical bulk datasets.

Current deconvolution methodologies are broadly stratified into reference-free and reference-based approaches. Reference-free methods, such as TOAST [6] and xCell [7], attempt to disentangle mixtures without external signatures but often suffer from lower resolution and stability. In contrast, reference-based strategies, which utilize expression signatures derived from scRNA-seq or sorted cell populations, generally yield superior performance [8]. Among reference-based approaches, traditional statistical learning methods such as CIBERSORTx [9] and BayesPrism [10] typically assume linear relationships between gene expression and cell proportions, assigning gene weights through optimization procedures [11]. However, gene expression in biological systems is inherently stochastic and non-linear, influenced by confounding factors such as cellular interactions, metabolic states, and batch effects. Recognizing this limitation, deep learning methods have emerged to address these complexities. Methods like Scaden [12] and TAPE [13] employ deep neural networks to approximate the complex, non-linear mapping between gene expression profiles and cell fractions, demonstrating significantly improved robustness against the technical noise inherent in transcriptomic data.

Despite the predictive gains achieved by deep learning, mainstream deconvolution frameworks face a critical interpretability bottleneck: they operate primarily as data-driven “black boxes.” Standard deep neural networks treat the transcriptome as an unstructured vector space, implicitly assuming gene independence or relying solely on latent statistical covariances. This “tabula rasa” learning approach fundamentally disregards the hierarchical architecture of biological systems, where gene expression is orchestrated by tightly regulated signaling cascades and interaction networks. By ignoring these inductive biases, purely data-driven models are prone to overfitting technical artifacts, learning the noise of the sequencing platform rather than the signal of cellular identity. Although recent attempts, such as DeSide [14], have sought to incorporate pathway information, they often rely on rudimentary integration strategies, such as element-wise multiplication of expression profiles with pathway annotations. Such simplified treatments fail to capture the synergistic interactions and directional logic embedded in biological networks, resulting in a suboptimal utilization of prior knowledge. Furthermore, the opacity of these “black box” models creates a credibility gap; without a transparent decision-making process, clinicians and biologists are unable to verify whether a model’s predictions are driven by known marker genes or spurious correlations, severely limiting their utility in mechanistic research.

Protein-protein interaction (PPI) networks provide a complementary layer of prior biological knowledge to pathway-based annotations [15]. While pathways typically summarize coordinated gene sets involved in specific biological processes, PPI networks describe the physical or functional interactions between proteins, thereby capturing the modular and hierarchical organization of cellular systems [16]. Integrating PPIs into computational models has been shown to improve robustness by encouraging genes to be interpreted in the context of their interaction partners rather than in isolation, which can help reduce overfitting to spurious, dataset‑specific correlations [17–19].

To address these fundamental challenges, we introduce iDCF (**I**nterpretable **D**econvolution of **C**ell **F**ractions), a biology-informed reference-based deep learning framework designed to achieve accurate, robust, and transparent estimation of cell-type proportions. iDCF relies on scRNA-seq–derived cell-type signatures as references to simulate pseudo-bulk training data for supervised deconvolution. In addition to these data-driven inputs, iDCF incorporates a protein–protein interaction (PPI) network derived from the STRING database and curated pathway annotations from MSigDB as global biological priors that constrain and guide the deconvolution process. By embedding bulk expression profiles into this PPI- and pathway-informed representation space, iDCF can preferentially leverage interactions supported by experimental or curated evidence and down-weight noisy, unstructured signals.

Unlike conventional models that rely solely on data fitting, iDCF adopts a novel dual-stream architecture that explicitly synergizes data-driven feature extraction with biologically informed constraints. The first stream employs a standard deep neural network (DNN) to capture high-dimensional latent patterns from the expression data. The second stream features a specialized knowledge-based sparse neural network (KSNN), which is structurally engineered using a “knowledge mask” derived from biological pathways and PPI networks. In this design, the links between neurons are not fully connected but are strictly defined by known biological interactions. Pathway information constrains functional relationships among genes, while PPI networks facilitate the identification of key regulatory nodes and hubs. By restricting network connectivity to established biological relationships, the KSNN compels the model to focus on extracting biologically meaningful, knowledge-driven features.

Crucially, iDCF transcends the traditional trade-off between accuracy and interpretability. We integrate the SHapley Additive exPlanations (SHAP) [20] framework directly into the model’s inference pipeline, transforming it from an opaque predictor into a transparent analytical tool. This allows for the precise quantification of how specific genes and pathway-derived features contribute to the estimated proportion of each cell type, enabling researchers to cross-validate model predictions against biological intuition. We conducted extensive benchmarking of iDCF across diverse biological contexts, including peripheral blood mononuclear cells (PBMCs), brain tissue from Alzheimer’s disease patients, and complex solid tumors. Our results demonstrate that iDCF achieves top-tier accuracy at both sample and cell-type levels and exhibits strong generalization capabilities across cross-platform datasets. Ultimately, by bridging the gap between data-driven deep learning and biological prior knowledge, iDCF offers a transparent and robust framework for cellular deconvolution, facilitating deeper insights into the complex cellular dynamics of tissue microenvironments.

## Results

### Overview of the iDCF framework

**Fig 1:**
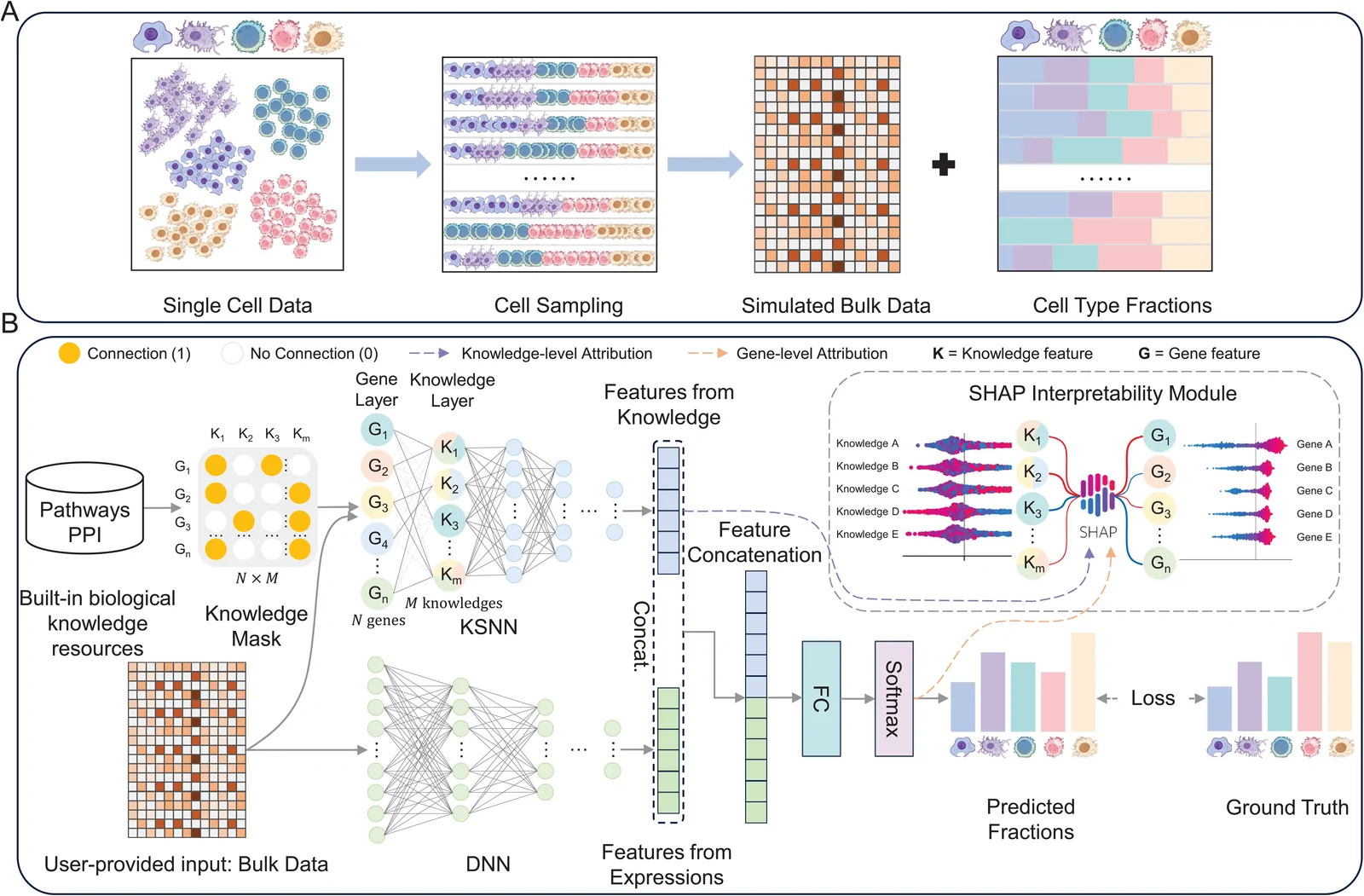
Workflow and architecture of the iDCF framework for interpretable cell deconvolution. **(A)** Construction of the pseudo-bulk training cohort. ScRNA-seq data serves as the source for generating pseudo-bulk samples through a randomized in silico cell sampling strategy. These simulated mixtures are assigned ground truth cell-type proportions to supervise model training. **(B)** The dual-stream neural network architecture. The model integrates transcriptomic data with biological priors through two parallel streams: (1) Data-driven stream (DNN): Processes bulk expression vectors through fully connected layers to extract high-dimensional latent features. (2) Knowledge-driven stream (KSNN): Utilizes a “knowledge mask” to impose structural constraints on a sparse neural network. Let *N* denote the number of input genes and *M* the number of knowledge features (pathways and PPI nodes). The knowledge mask is an N×M matrix. Connections between the “gene layer” and “knowledge layer” (where *G* and *K* denote gene and knowledge features, respectively) are strictly limited to biologically established interactions (e.g., gene-pathway or PPI associations), filtering out spurious correlations. The PPI network (from STRING) and pathway annotations (from MSigDB) are provided as integrated, built-in resources within the iDCF framework and do not need to be supplied by the user. Deep features from both streams are concatenated and passed through a fusion layer and softmax classifier to minimize the loss against ground truth. The “SHAP interpretability module” dissects the model’s decision logic at two distinct levels to reveal the biological basis of predictions: Knowledge-level attribution (Left panel): Interrogates the knowledge feature vector output by the KSNN stream. By computing directional SHAP values for these intermediate features, this analysis ranks biological modules based on their influence on the predicted cell-type proportions, revealing whether each module exerts a positive or negative effect. Gene-level attribution (Right panel): Computes directional SHAP values for individual gene inputs relative to the model’s final output layer. This reveals whether specific gene expression levels exert a positive or negative influence on the estimation of a given cell type. **Image attribution:** Fig 1 incorporates cell icons from the NIH BioArt Source (BioArt IDs 111, 376, 409, and 508; Public Domain) and one graphical element from https://doi.org/10.5281/zenodo.3926101 (CC BY 4.0).

To achieve accurate and transparent cell-type deconvolution, we developed iDCF, a biology-informed deep learning framework. As illustrated in Fig 1, the workflow proceeds in two phases. First, we construct a comprehensive training cohort of pseudo-bulk samples derived from scRNA-seq data (Fig 1A). By employing a simulated cell sampling strategy, we generate mixtures with variable but known cell-type proportions, establishing a high-fidelity ground truth for model supervision. Detailed procedures for pseudo-bulk data construction are provided in the Methods section.

The core of iDCF lies in its dual-stream architecture (Fig 1B), designed to synergize data-driven learning with biological priors. The first stream utilizes a standard DNN to capture latent high-dimensional patterns from gene expression data end-to-end. In parallel, the second stream features a KSNN, explicitly structured by a “knowledge mask” encoding pathways and PPI networks. Unlike fully connected layers, the KSNN restricts connectivity to biologically validated interactions, compelling the model to extract features that are biologically meaningful rather than purely statistical. Notably, iDCF takes bulk expression data as the primary user-provided input. The PPI network and biological pathway are not user inputs; instead, they are included as integrated, pre-compiled resources (STRING PPI and MSigDB pathways) within the iDCF framework. Detailed architectural information of the KSNN and DNN modules is provided in S5 Fig.

These complementary feature sets, latent patterns from the DNN and structured biological features from the KSNN, are fused via a fully connected layer before a softmax output layer predicts cell-type proportions. The model is optimized by minimizing the divergence between predicted and ground truth fractions. Finally, to bridge the gap between computational inference and biological mechanism, we integrated a SHAP-based interpretability module. Unlike purely post-hoc explanations of unstructured models, this framework leverages iDCF’s knowledge architecture to provide biologically aligned feature attribution. By quantifying the contribution of specific genes and functional nodes to the final prediction, it enables the rigorous verification of decision logic against established biological intuition.

### Performance on independent pseudo-bulk datasets

To validate the robustness and generalization capability of iDCF, we benchmarked it against four deep learning algorithms, including DeSide [14], scpDeconv [21], TAPE [13] (in both TAPE-O and TAPE-H modes), and Scaden [12], and three established statistical methods (ReDeconv [22], BayesPrism [10], and CIBERSORTx [9]). The models were trained on pseudo-bulk data derived from the PBMC8k dataset [23], while statistical approaches were applied directly using their standard protocols. To rigorously assess performance on unseen data, we generated 500 pseudo-bulk test samples from two independent cohorts: PBMC6k [24] and DonorA [25], which introduce distinct donor-specific biological variations and experimental batch effects.

**Fig 2:**
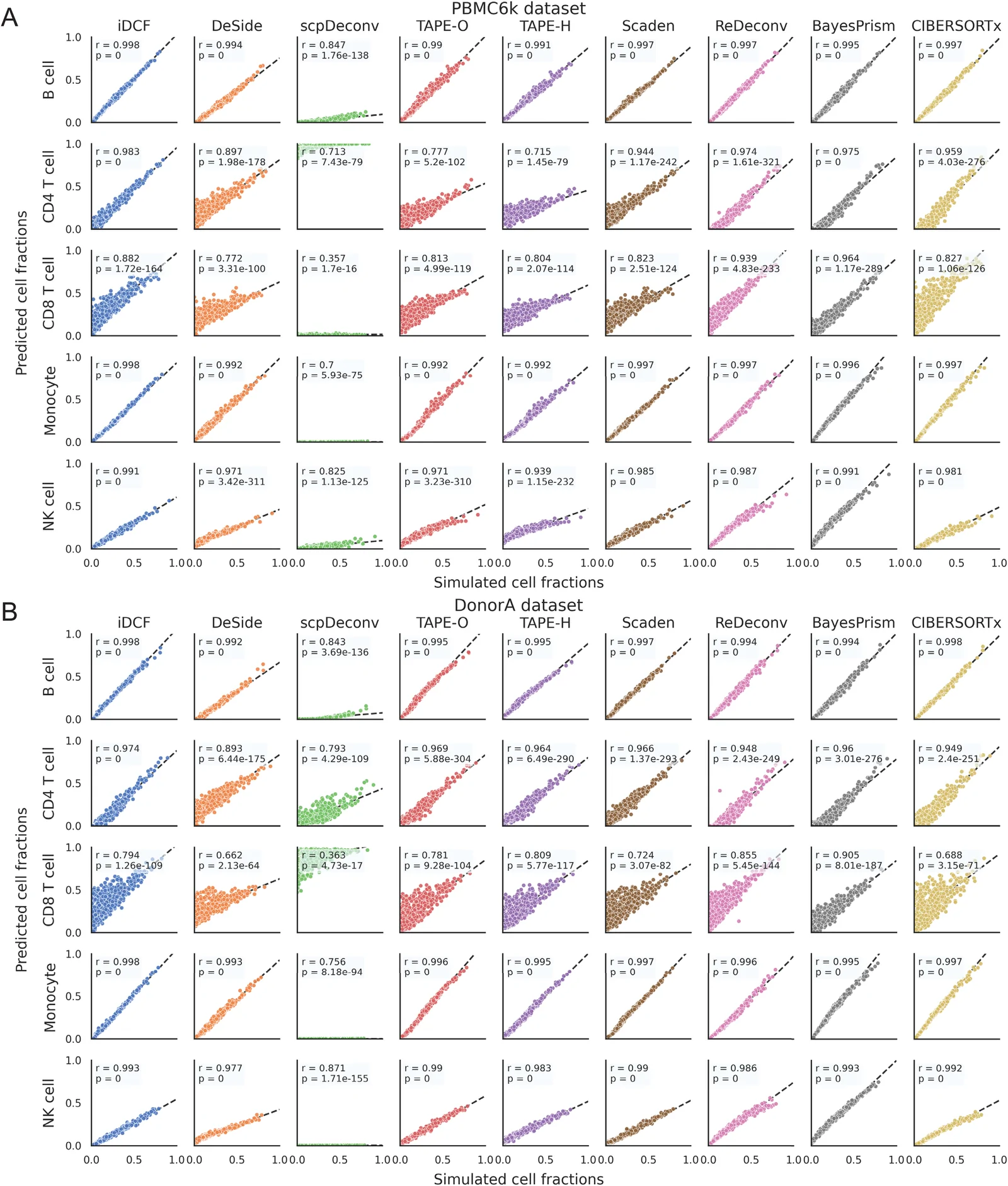
Performance benchmarking of iDCF on independent pseudo-bulk cohorts. Scatter plots illustrating the concordance between predicted cell proportions (*y*-axis) and ground truth fractions (*x*-axis) across five major cell types. The benchmark compares iDCF (leftmost column) against seven state-of-the-art deconvolution methods, grouped into reference-based deep learning methods (DeSide, scpDeconv, TAPE, Scaden) and reference-based statistical methods (ReDeconv, BayesPrism, CIBERSORTx). **(A)** Performance on the PBMC6k dataset. **(B)** Performance on the DonorA dataset, representing a distinct experimental batch. Numerical annotations in each panel indicate the Pearson correlation coefficient (*r*) and the corresponding *P*-value. The black dashed line represents the linear regression fit. Note that iDCF consistently maintains high linearity and tight clustering around the diagonal across all cell types, particularly for the challenging T cell subtypes, demonstrating superior generalization compared to other deep learning and statistical baselines.

As illustrated in Fig 2, iDCF exhibited superior performance across both independent test sets, achieving the highest median Pearson correlation coefficient (*r*) across all evaluated cell types (summarized in S5 Table). In addition, a summarizing heatmap of *r* values between different methods is provided in S6 Fig to further illustrate the comparative performance. Among all deep learning-based methods, iDCF achieved the highest deconvolution performance. When compared to statistical approaches, iDCF performed competitively, performing comparably to ReDeconv and BayesPrism for most cell types and achieving the highest *r* for B cells, monocytes, and NK cells.

Notably, a core strength of iDCF lies in its fine-grained resolution of highly homologous cell populations. Distinguishing between phenotypically similar subsets, such as CD4 and CD8 T cells, represents a notorious challenge in deconvolution due to high transcriptional collinearity. iDCF demonstrated strong performance in resolving these nuances. This performance advantage suggests that the KSNN stream successfully captures the subtle, pathway-level regulatory divergences, that distinguish these lineages, whereas baseline models are confounded by their global expression similarities.

These results provide compelling evidence that iDCF successfully extracts transferable, identity-defining biological features rather than overfitting to dataset-specific technical noise. This robustness against batch effects establishes a solid foundation for applying iDCF to more complex, real-world bulk transcriptomic data.

### Deconvolution of bulk data and comparison with flow cytometry

To rigorously evaluate iDCF in real-world scenarios, we benchmarked its performance against flow cytometry ground truth across eight independent PBMC bulk transcriptomic datasets. These datasets encompass diverse experimental conditions and sequencing platforms, serving as a stringent test for generalization. Using the PBMC8k scRNA-seq dataset [23] as the sole reference, we assessed deconvolution fidelity using the Lin’s concordance correlation coefficient (CCC) and mean absolute error (MAE).

**Fig 3:**
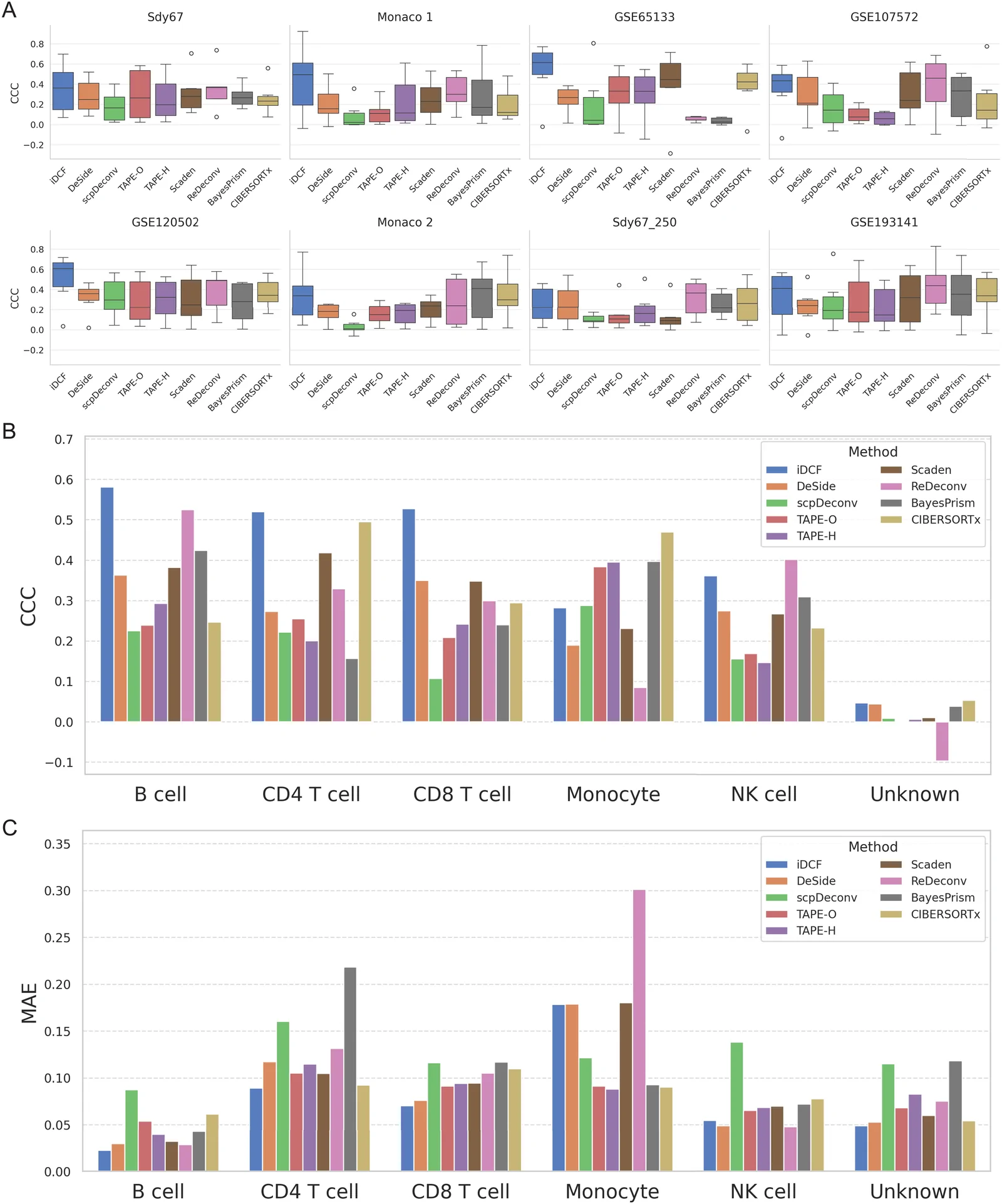
Benchmarking deconvolution performance against flow cytometry ground truth across real-world cohorts. **(A)** Distribution of Lin’s concordance correlation coefficients (CCC) for iDCF and seven competing methods, grouped into reference-based deep learning methods (DeSide, scpDeconv, TAPE, Scaden) and reference-based statistical methods (ReDeconv, BayesPrism, CIBERSORTx) across eight independent PBMC bulk datasets. The box plots summarize the agreement between predicted cell proportions and flow cytometry measurements. Center lines indicate medians; box limits indicate the 25th and 75th percentiles. iDCF consistently achieves high median CCC with low variance. **(B, C)** Method performance stratified by individual cell types. Bar plots illustrate the (b) average CCC (higher is better) and (c) mean absolute error (MAE, lower is better) across all eight datasets for six major immune cell populations. Note that iDCF dominates in lymphocyte quantification (B cells, CD4 T, CD8 **T)**, demonstrating superior resolution for T cell subsets compared to both deep learning and statistical baselines.

iDCF demonstrated exceptional cross-cohort robustness. As shown in Fig 3A, iDCF achieved the highest median CCC in five out of eight datasets (including Sdy67, Monaco 1, GSE65133, GSE107572, and GSE120502), indicating superior alignment with flow cytometry measurements. Even in datasets where it did not rank first, iDCF remained highly competitive, with CCCs significantly outperforming most other methods. In contrast, methods such as scpDeconv and ReDeconv displayed significant performance volatility across varying batches. This consistency was further corroborated by the MAE metric (Figs 3C and S1A), where iDCF yielded the lowest error rates in the majority of cohorts, underscoring its reliability for clinical-grade applications.

A granular assessment at the cell-type level (Fig 3B and 3C) revealed iDCF’s distinct advantage in quantifying lymphocyte subpopulations. Specifically, iDCF achieved the highest average CCC and lowest MAE for B cells, CD4 T cells, and CD8 T cells (with the specific average CCC and MAE values provided in S6 Table), effectively resolving the challenge of differentiating highly homologous T cell subsets in bulk mixtures. For NK cells, iDCF performed exceptionally well, ranking second only to ReDeconv. Regarding monocytes, while CIBERSORTx exhibited a marginal performance advantage, iDCF maintained robust accuracy, demonstrating a balanced trade-off between sensitivity and specificity across the full cellular spectrum.

### Genetic interpretation of iDCF

**Fig 4:**
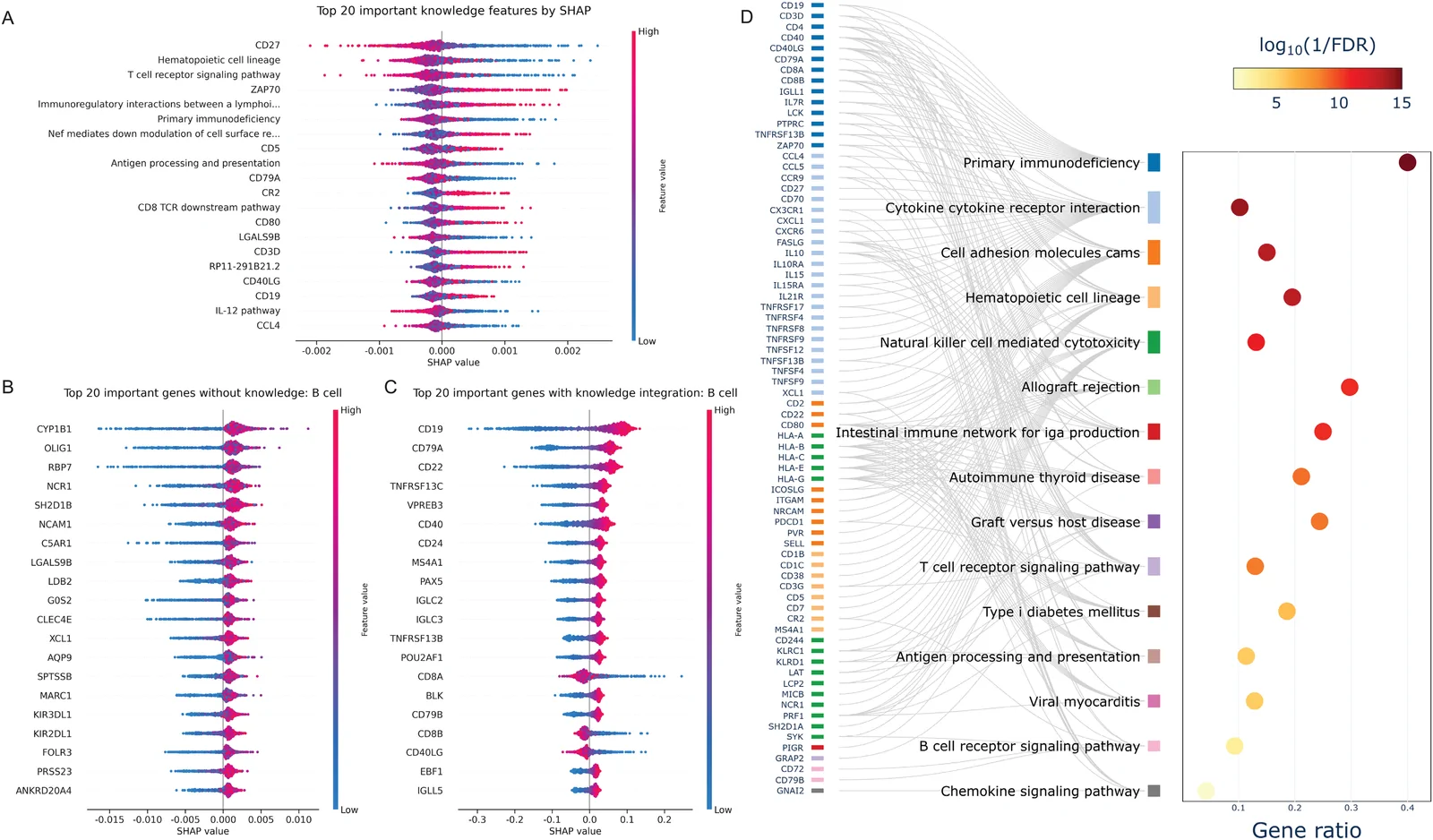
Unveiling the molecular decision logic of iDCF via SHAP interpretability analysis. **(A)** SHAP summary plot displaying the top 20 biological knowledge features contributing most to model predictions. Each point represents a sample, with color indicating feature value magnitude (red for high, blue for low), and its horizontal position on the x-axis (SHAP value) indicating both the direction and magnitude of the feature’s influence on the prediction. Due to space constraints, some pathway names in the figure are truncated with ellipses. The full names for the truncated entries are as follows: “Immunoregulatory interactions between a lymphoi...” stands for “Immunoregulatory interactions between a lymphoid and a non lymphoid cell”; “Nef mediates down modulation of cell surface...” stands for “Nef mediates down modulation of cell surface receptors by recruiting them to clathrin adapters.” **(B)** SHAP summary plot of the top 20 genes contributing most to B cell proportion predictions when iDCF operates without biological knowledge integration. **(C)** SHAP summary plot of the top 20 genes contributing most to B cell proportion predictions in the complete iDCF model with integrated biological knowledge. iDCF (c) exhibits SHAP values an order of magnitude larger than the baseline **(b)**, reflecting that biological knowledge integration accentuates the predictive impact of canonical markers (e.g., CD19, CD79A, PAX5) by concentrating model focus, whereas the baseline distributes weights across scattered statistical noise (e.g., CYP1B1). **(D)** KEGG functional enrichment analysis of genes contained within the top 200 highest-contributing knowledge features. The figure combines a Sankey diagram (left) and a dot plot (right) to visualize significantly enriched immune-related pathways. The Sankey diagram illustrates the relationship between specific genes (left column) and the pathways they are involved in (middle column). The dot plot quantifies the enrichment results, where the x-axis represents the Gene Ratio, and the color of the dots indicates the statistical significance (scaled as *log*_10_(1/*FDR*), where FDR, false discovery rate, is used as the adjusted P-value).

To transcend the “black box” limitations of traditional deep learning, we conducted a comprehensive interpretability analysis using the SHAP framework to dissect the molecular logic governing iDCF’s predictions. Using the PBMC8k dataset as a reference and the independent Monaco 1 dataset as a case study, we probed the model’s transparency across three granular levels. First, we quantified the contribution of biological knowledge features within the KSNN, identifying and visualizing the top 20 most influential features (where pathways are denoted by name and PPI nodes by gene symbols, as shown in Fig 4A). In these summary plots, the x-axis represents the SHAP value, indicating the direction and magnitude of each feature’s effect on the predicted cell fraction (positive values increase, whereas negative values decrease the prediction). At the same time, the dot color encodes the relative feature value in each sample, with red denoting higher values and blue denoting lower values. Second, to pinpoint specific molecular drivers, we compared the top 20 genes contributing to B cell prediction in the full iDCF model versus a baseline data-driven model (Fig 4B and 4C). Finally, to systematically map these learned features to broad biological functions, we performed Kyoto Encyclopedia of Genes and Genomes (KEGG) pathway enrichment analysis on the constituent genes of the top 200 most influential knowledge nodes (Fig 4D).

This multi-level dissection revealed that iDCF’s decision-making is strictly governed by established immunological principles. As shown in Fig 4A, the top 20 knowledge features prioritizing the model’s output were heavily enriched for immune-specific functions. Canonical pathways, including *Hematopoietic cell lineage*, *T cell receptor signaling*, and *Antigen processing and presentation*, ranked among the highest contributors. Furthermore, the model correctly identified key linchpin genes within the network, such as CD27, ZAP70 (a critical T-cell signaling node), CD5, and CD79A (a B cell antigen receptor component) [26–31]. These findings provide compelling evidence that the “knowledge mask” within iDCF effectively constrains the neural network, compelling it to leverage biologically valid signals to guide accurate cellular deconvolution.

We focused on B cells to conduct a comparative case study between the full iDCF framework and a baseline unconstrained model. In the absence of knowledge guidance (Fig 4B), the baseline model exhibited critical flaws in feature attribution. It relied heavily on spurious correlations, assigning top rank to genes such as CYP1B1, OLIG1, and RBP7, features biologically irrelevant to B cell identity. This indicates that the unconstrained model learned to exploit dataset-specific noise rather than capturing true biological signals. Second, the decision logic appeared stochastic; SHAP values clustered tightly around zero (range: -0.015 to 0.010), implying that the model failed to identify potent predictors and instead depended on the aggregation of numerous weak, noisy signals. Such behavior fundamentally undermines prediction credibility.

In stark contrast, the knowledge-integrated iDCF model correctly triangulated B cell identity through canonical linchpin markers (CD19, CD79A, PAX5) and functional hubs (B cell receptor signaling)(Fig 4C). Specifically, the canonical B cell marker CD19 shows a consistent positive correlation, where higher expression (red dots) corresponds to positive SHAP values that elevate the predicted B cell proportion. Conversely, the non-target T cell marker CD8A demonstrates an exclusive effect; its higher expression yields negative SHAP values, reflecting the compositional crowding out of B cells by T cells in the mixture. Beyond markers, it captured functional logic, identifying B cell receptor components (VPREB3, IGLC2) and survival signaling molecules (CD40, TNFRSF13C) as top predictors [32–34]. This biological alignment was accompanied by a notable expansion in feature influence. The range of SHAP values increased by over an order of magnitude (approximately -0.3 to 0.2) compared to the baseline. This expansion indicates that the knowledge mask effectively concentrates the model’s focus on key biological drivers rather than scattering weights across statistical noise, thereby amplifying the predictive impact of primary features.

Systematic validation via KEGG enrichment analysis (Fig 4D) further corroborated this logic. The genes comprising the top 200 knowledge features were significantly enriched in pathways essential for immunity, such as *Primary immunodeficiency*, *Hematopoietic cell lineage*, and the *B cell receptor signaling pathway* [26]. Collectively, these results demonstrate that iDCF transcends the opacity of standard deep learning. By enforcing structural constraints based on biological priors, iDCF ensures that predictions are not only statistically accurate but also mechanistically transparent and robust.

### Clinical interpretation of iDCF

To evaluate whether iDCF can corroborate biological findings using clinical data, we analyzed five datasets with associated clinical information.

#### Alzheimer’s disease.

**Fig 5:**
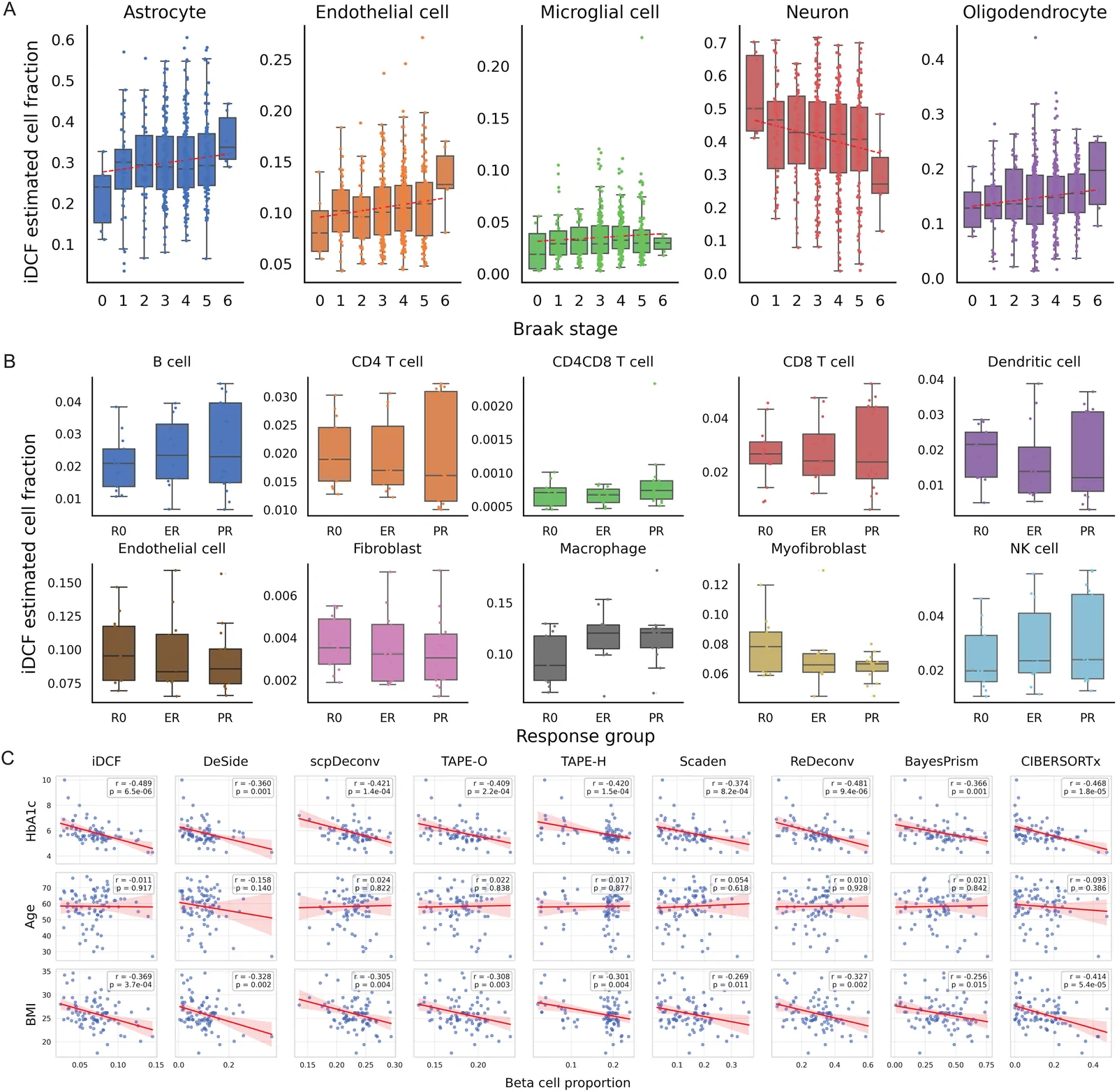
iDCF validation of known biological facts across multiple clinical datasets. (A) iDCF-inferred cellular dynamics across Alzheimer’s disease Braak stages in the ROSMAP cohort. Boxplots depict the proportion changes of five major brain cell types. The model captures a progressive loss of neurons and a concurrent rise in astrocytes, while microglia exhibit non-linear dynamics, expanding with disease severity but notably declining at the terminal stage (Braak stage 6). (B) Immune landscape stratification in high-grade serous ovarian cancer (HGSC) associated with therapeutic response. Patients with optimal surgical outcomes (R0) display a distinct immune signature: significantly lower macrophage abundance and higher CD8 T cell infiltration compared to those with poor (PR) or excellent (ER) response to neoadjuvant chemotherapy. (C) Association between beta-cell mass and metabolic phenotypes in type 2 diabetes (GSE50244). Scatter plots compare the correlation of predicted beta-cell proportions against HbA1c (top), Age (middle), and BMI (bottom) across nine methods. iDCF (leftmost column) reveals the strongest negative correlation with HbA1c (r=−0.489) and BMI (r=−0.369), accurately reflecting beta-cell dysfunction.

To demonstrate the translational utility of iDCF in reconstructing complex tissue pathologies, we first analyzed the religious orders study and memory and aging Project (ROSMAP) dataset [35], comprising bulk RNA-seq profiles from the dorsolateral prefrontal cortex of Alzheimer’s disease (AD) patients and controls. Leveraging a reference atlas of the human prefrontal cortex [36–39], we benchmarked performance on a subset of 41 samples with matched immunohistochemistry (IHC) ground truth. iDCF achieved the highest median CCC among all methods (S1B and S1C Fig), effectively bridging the gap between transcriptomic inference and histological reality. This result critically validates iDCF’s robust generalization capability, extending its applicability from peripheral blood to complex solid tissues such as the brain. Crucially, when applied to the broader cohort (493 samples) stratified by Braak staging, iDCF accurately recapitulated the cellular trajectory of AD progression (Fig 5A). It revealed a quantitative signature of neurodegeneration characterized by the progressive loss of neurons [40], mirrored by a compensatory rise in astrocyte proportions indicative of reactive astrogliosis [41,42]. Furthermore, iDCF captured the sophisticated, non-linear dynamics of microglia: their proportions expanded with increasing disease severity but notably declined at the terminal stage (Braak stage 6). This observation aligns with established models of microglial activation and subsequent exhaustion or dystrophy in late-stage neurodegeneration [43–45], highlighting iDCF’s sensitivity to subtle, stage-dependent pathological shifts.

#### High-grade serous ovarian cancer.

Extending our analysis to the complex solid tumor microenvironment, we applied iDCF to investigate high-grade serous ovarian cancer (HGSC), a malignancy characterized by significant immune heterogeneity. We utilized a single-cell reference atlas [46] to deconvolute bulk RNA-seq profiles from a clinical cohort of 41 primary HGSC samples [47,48]. To assess clinical relevance, patients were stratified into three distinct therapeutic outcome groups: those achieving optimal surgical debulking with no residual disease (R0), and those receiving neoadjuvant chemotherapy (NACT) with either poor response (PR) or excellent response (ER). iDCF successfully stratified the patient cohort into distinct immunological archetypes (Fig 5B). The R0 group exhibited an immunologically active profile enriched for cytotoxic CD8 T cells and depleted of immunosuppressive macrophages, consistent with the improved chemo-sensitivity and surgical outcomes associated with immunologically hot tumor microenvironments. This distribution aligns with the observations of Lee et al., who reported macrophage enrichment in non-optimal outcomes. Conversely, iDCF identified a significant enrichment of CD8 T cells in the R0 group relative to the NACT groups. By accurately capturing these opposing trends, macrophage exclusion and CD8 T cell infiltration, iDCF validated its ability to resolve the delicate balance of the immune milieu associated with ovarian cancer prognosis [47].

#### Diabetes mellitus.

To validate iDCF in the context of metabolic disorders, we analyzed the cellular composition of human pancreatic islets using the GSE50244 bulk RNA-seq dataset [49], comprising samples from patients with type 2 diabetes (T2D) and healthy controls. Leveraging a pancreas-specific single-cell reference [50] (encompassing alpha, beta, delta, gamma, acinar, and ductal cells), we investigated the link between cellular heterogeneity and clinical metabolic phenotypes in 77 donors with matched Hemoglobin A1c (HbA1c) data. iDCF accurately captured the hallmark pathology of T2D: the depletion of functional beta-cell mass. As illustrated in Fig 5C, iDCF inferred beta-cell proportions exhibited the strongest negative correlation with HbA1c levels among all benchmarked methods (r=−0.489, $p=6.5\times 10^{-6}$). This robust inverse relationship quantitatively links the loss of insulin-producing cells to the worsening of long-term glycemic control. Additionally, iDCF revealed a significant negative association between beta-cell abundance and body mass index (BMI) (r=−0.369, $p=3.7\times 10^{-4}$), reflecting the impact of metabolic stress on islet integrity. Importantly, no significant correlation was found between age and beta-cell proportion across any method, confirming that the observed associations are specific to disease pathology rather than confounding demographic factors. These results indicate that iDCF’s predictions accurately reflect the key pathophysiological state of T2D.

#### SARS-CoV-2 infection of pancreatic islets.

To validate iDCF’s sensitivity to acute viral pathogenesis and pharmacological response, we analyzed an ex vivo infection model using the GSE159717 bulk RNA-seq dataset [51]. This cohort comprised human pancreatic islets across three conditions: uninfected controls, samples infected with SARS-CoV-2, and infected samples treated with the antiviral drug remdesivir. Using the same healthy pancreas reference as above (GSE84133, selecting alpha, beta, delta, and gamma cells), we tracked the compositional dynamics of endocrine cells. iDCF precisely mapped the infection-induced trajectory consistent with known cytopathic effects (S2A Fig). Previous studies have established that SARS-CoV-2 tropism for beta-cells leads to their dysfunction and loss [51]. iDCF accurately captured this phenomenon, revealing a marked depletion of beta-cell proportions in the infected group. Most importantly, iDCF uniquely quantified the restorative efficacy of remdesivir. While other methods detected general trends, iDCF demonstrated superior sensitivity in reflecting the therapeutic outcome: the predicted beta-cell proportions in the remdesivir-treated group rebounded to levels nearly indistinguishable from uninfected controls. This finding underscores iDCF’s capacity to serve as a high-fidelity tool for evaluating tissue-level recovery in drug screening applications.

To address the sample-size limitation, we additionally evaluated an independent dataset (GSE165890 [52]) comprising 12 primary human pancreatic islet samples (6 uninfected controls and 6 SARS-CoV-2–infected). A one-tailed *t*-test was used to assess differences in the predicted β-cell proportions between groups. Notably, iDCF, together with DeSide, TAPE, and ReDeconv, robustly detected the post-infection depletion of β cells (*p* < 0.01). In contrast, Scaden and BayesPrism showed only weaker significance (*p* < 0.05), while scpDeconv and CIBERSORTx did not reveal any significant difference (S2B Fig and S7 Table).

#### Small extracellular vesicles.

Finally, we challenged iDCF to decode the cryptic signals of small extracellular vesicles (sEVs) in colorectal cancer (CRC). Recognizing that sEV-derived RNA constitutes a trace fraction of the bulk transcriptome, rendering precise proportion estimation challenging, we pivoted our analysis to qualitatively probe the functional signature prioritized by the model. We leveraged the SHAP interpretability module to dissect the transcriptomic signature that the model prioritizes when distinguishing sEV-associated signals. To establish a high-fidelity ground truth, we utilized the single-cell cohort from Lee et al. [53] and applied the specialized algorithm sEVtras [54] to rigorously annotate individual profiles as either “sEV” or “Others.” Using this refined reference, we interrogated the bulk RNA-seq profiles of 483 CRC tumors and 41 normal controls from the The Cancer Genome Atlas (TCGA) cohort [55].

This analysis uncovered a fundamental “functional reprogramming” of the vesicular secretome in the tumor microenvironment (S3 Fig). While shared features (e.g., TTN and microenvironment sensing pathways) highlighted the conserved role of sEVs in structural maintenance, the divergent features were biologically revealing [56,57]. In normal tissues, the model prioritized features like REG3A and TCF7, genes intrinsic to mucosal defense and epithelial stemness [58,59], reflecting a homeostatic signature geared towards tissue repair. In stark contrast, tumor-derived sEVs exhibited a shift towards oncogenic and immunosuppressive functions. iDCF identified key drivers associated with dedifferentiation (keratinization pathways) and, notably, immune evasion markers such as KLRD1 and PDCD1 (encoding PD-1) [60]. This suggests that CRC cells may hijack vesicle trafficking pathways to export immune checkpoints or modulate the immune landscape remotely. Collectively, these findings demonstrate that iDCF’s interpretability framework can look beyond cellular abundance to reveal how the functional state of subcellular mediators is rewired during tumor progression.

### Contributions of biological pathways and PPI

To validate the architectural rationale of iDCF, we performed a systematic ablation study to disentangle the contributions of its two knowledge-driven components (biological pathways and PPI) and the data-driven DNN module. Specifically, we evaluated five model configurations: the full iDCF model; three knowledge-deficient variants—iDCF without pathways (-Path), iDCF without PPI (-PPI), and iDCF without both pathways and PPI (-Path&PPI); and a knowledge-only baseline, iDCF without the DNN module (-DNN).

**Fig 6:**
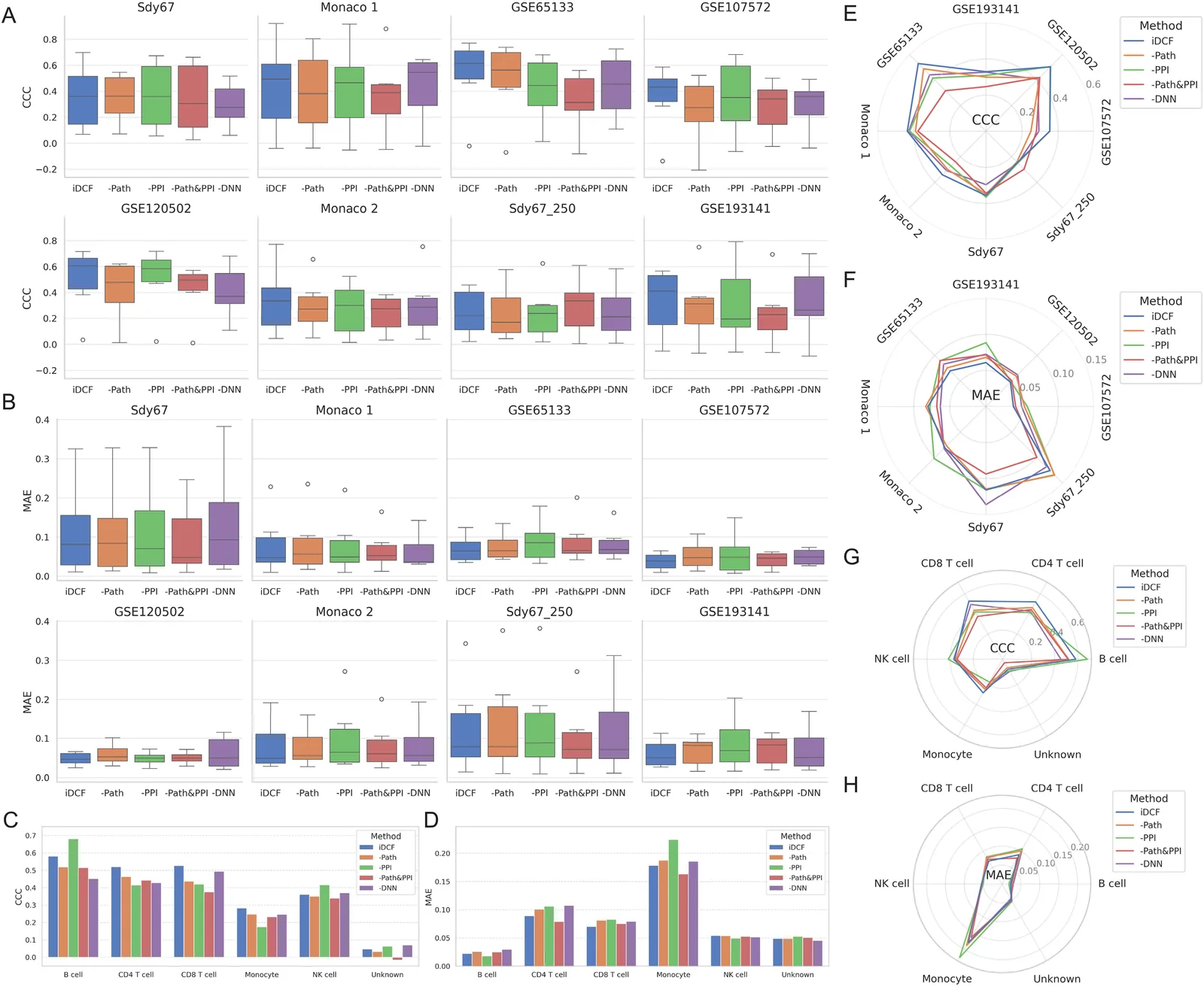
Ablation analysis of different knowledge modules and network components in iDCF. Performance comparison of five model configurations: the complete iDCF; variants lacking specific prior streams, including iDCF w/o Pathways (denoted as -Path) and iDCF w/o PPI (denoted as -PPI); the DNN-only baseline lacking all biological priors, iDCF w/o both pathways & PPI (denoted as -Path&PPI); and the knowledge-only variant lacking the standard DNN stream, iDCF w/o DNN (denoted as -DNN). **(A, B)** Distribution of **(A)** CCC and **(B)** MAE across eight PBMC datasets. While the contribution of pathways versus PPIs varies by dataset, the full iDCF model consistently achieves the most robust performance, outperforming both the -Path&PPI and -DNN configurations on the majority of cohorts. **(C, D)** Cell-type specific accuracy metrics. Bar charts illustrate that integrating both knowledge streams ensures optimal deconvolution for the majority of cell types. **(E–H)** Multi-dimensional visualization of the comprehensive performance advantage. Radar charts map the performance across (E, F) individual datasets and (G, H) specific cell types.

The results revealed a distinct complementarity between the two knowledge sources. As illustrated in Fig 6A and 6B, while integrating either pathways or PPIs individually yielded performance gains over the baseline (-Path&PPI), their relative contributions exhibited significant context-dependence across datasets. In some cohorts, pathway information proved more critical, whereas in others, PPI drove the primary improvements. Notably, the -DNN variant (lacking the DNN module) underperformed relative to the full iDCF model on most datasets, underscoring the essential role of the neural network in integrating and refining knowledge-based features. Crucially, the full iDCF model consistently achieved the most robust and optimal performance across the majority of test scenarios. This confirms that iDCF does not merely accumulate data; rather, it effectively synergizes the functional constraints of pathways with the interaction logic of PPIs, mitigating the limitations of relying on a single knowledge modality.

This synergistic advantage was further evident at the cellular resolution (Fig 6C and 6D). While different cell types demonstrated varying dependencies on pathway versus PPI features, the full model maintained optimal or near-optimal accuracy across the entire cellular spectrum. The radar charts (Fig 6E–6H) provide a multi-dimensional visualization of this comprehensive performance advantage, demonstrating that the integration of dual-stream knowledge is essential for achieving stable, high-fidelity deconvolution across diverse biological contexts.

## Materials and methods

### Datasets and preprocessing

#### scRNA-seq datasets and preprocessing.

All scRNA-seq datasets utilized as references in this study are publicly available; detailed metadata are summarized in S1 Table. The PBMC cohorts, comprising PBMC8k, PBMC6k, and DonorA, were obtained from the 10x Genomics repository. The human prefrontal cortex dataset, used for deconvolution of the ROSMAP cohort, was curated by Song et al. [36] and retrieved from the STAB database (https://mai.fudan.edu.cn/stab/). Reference datasets for HGSC and pancreatic islet analyses were downloaded from the Gene Expression Omnibus (GEO) under accession numbers GSE232314 [46] and GSE84133 [50], respectively. The CRC reference dataset was obtained from ArrayExpress under accession number E-MTAB-8410 [53].

Data preprocessing and quality control were conducted using Scanpy [61]. For the PBMC datasets, low-quality cells expressing fewer than 500 genes and genes detected in fewer than five cells were excluded. Automated cell annotation was performed using CellTypist [62], after which 18 predicted subtypes were consolidated into six major cell lineages (S2 Table). For the CRC dataset, raw expression matrices were processed using the sEV_recognizer module of sEVtras [54] to stratify profiles into “sEV” or “Other”; the score threshold (score_t) was set to 10, with all other parameters maintained at default values. For the remaining datasets (Brain, HGSC, and Islet), the original cell-type annotations provided by the respective source studies were retained.

#### Bulk RNA-seq datasets and preprocessing.

To rigorously evaluate model performance and conduct ablation studies, eight bulk RNA-seq datasets with ground truth cell proportions were utilized. The Sdy67 and Sdy67_250 datasets, originally generated by Zimmermann et al. [63], were sourced from the Scaden publication [12] and ImmPort (Accession: SDY67), respectively, with ground truths for the latter provided by Monaco et al. [64]. The Monaco 1 (GSE107011) and Monaco 2 (GSE107990) datasets [64] were downloaded from the Gene Expression Omnibus (GEO), with cell proportions extracted from the original supplementary materials. The datasets GSE65133 [65], GSE107572 [66], and GSE120502 [67] were similarly acquired from GEO, with ground truth data available on the respective sample detail pages. The GSE193141 dataset [68] was obtained with proportions provided in the original supplementary files. To ensure cross-dataset comparability, cell subtype annotations across all benchmarking cohorts were harmonized into six major cell lineages (S4 Table).

For real-world clinical validation, five distinct cohorts were employed. The ROSMAP dataset for Alzheimer’s disease research was obtained via Synapse (ID: syn3219045) [69]. From this cohort, two specific subsets were utilized: 41 samples with IHC-validated cell proportions (sourced from the TAPE study [13]) and 493 preprocessed samples containing Braak staging metadata (sourced from Patrick et al. [70]). The validation dataset for HGSC, originally generated by Lee et al. [47], was retrieved from the study by Guo et al. [48]. To assess metabolic disorders, the GSE50244 dataset [49] was downloaded from GEO, including sample-matched clinical indicators such as HbA1c. For the acute infection analysis, GSE159717 [51], comprising islet samples from two donors across three treatment conditions, was acquired from GEO. We additionally analyzed an independent SARS-CoV-2 dataset GSE165890 [52], consisting of 12 primary human pancreatic islet samples (6 controls and 6 infected). Finally, transcriptomic profiles for the sEV analysis were derived from TCGA colon adenocarcinoma (COAD) project [55], consisting of 483 tumor and 41 adjacent non-tumor samples. All bulk RNA-seq datasets described above are summarized in S3 Table. All source data underlying the main and supporting figures are provided in S1 Data.

### The iDCF framework

#### Definition of cell deconvolution.

The task of cell deconvolution is predicated on a linear mixture model, which assumes that the gene expression profile of a tissue sample containing multiple cell types (i.e., a “bulk” sample) can be approximated by the weighted sum of the gene expression profiles of its constituent cell types and their corresponding proportions. This relationship can be expressed in matrix form as:


$$\begin{array}{c} 𝐁=𝐅\cdot 𝐒\text{,} \end{array}$$ (1)

where $𝐁\in {\mathbb{R}}^{m\times g}$ denotes the input bulk expression matrix (*m* samples, *g* genes); $𝐅\in {\mathbb{R}}^{m\times c}$ represents the cell proportion matrix to be estimated (*c* cell types); and $𝐒\in {\mathbb{R}}^{c\times g}$ is the reference si*g*nature matrix derived from single-cell data.

Traditionally, deconvolution is treated as a constrained optimization problem to solve for 𝐅:


$$\begin{array}{c} \min_{𝐅}\|𝐁-𝐅\cdot 𝐒{\|}_{F}^{2}\;\text{s.t.}\;F_{ik}\geq 0,\;\sum_{k=1}^{c}F_{ik}=1\text{,} \end{array}$$ (2)

where *i* (1,...,m) denotes the sample index, *k* (1,...,c) denotes the cell type index, and $\|\cdot {\|}_{F}$ represents the Frobenius norm. The constraints ensure biological validity: estimated proportions must be non-negative and sum to one for each sample.

Unlike traditional approaches that solve this equation per sample, iDCF reframes deconvolution as a data-driven, non-linear regression task. The objective is to learn a mapping function $f_{\Theta }:{\mathbb{R}}^{g}\to \Delta^{c-1}$ that predicts the proportion vector $\hat{y}$ directly from a gene expression vector $𝐱\in {\mathbb{R}}^{1\times g}$:


$$\begin{array}{c} \hat{y}=f_{\Theta }(𝐱)\text{.} \end{array}$$ (3)

The architecture of $f_{\Theta }$ extracts features via two parallel streams. The first stream captures data-driven latent patterns ${𝐡}_{\text{exp}}$ using a standard DNN:


$$\begin{array}{c} {𝐡}_{\text{exp}}=\text{DNN}(𝐱)\text{.} \end{array}$$ (4)

The second stream extracts knowledge-guided features ${𝐡}_{\text{know}}$ via the KSNN:


$$\begin{array}{c} {𝐡}_{\text{know}}=\text{KSNN}(𝐱)=𝐱\left({𝐖}_{\text{know}}⊙𝐌\right)\text{,} \end{array}$$ (5)

where ${𝐖}_{\text{know}}\in {\mathbb{R}}^{g\times p}$ is the learnable weight matrix, $𝐌\in \{0,1{\}}^{g\times p}$ is the binary knowledge mask, and ⊙ denotes the Hadamard product. Here, *p* denotes the number of knowledge features.

Subsequently, the feature vectors from both streams are concatenated and projected to the output space via a fully connected layer with a Softmax activation:


$$\begin{array}{c} \hat{y}=\text{Softmax}\left([{𝐡}_{\text{exp}}∥{𝐡}_{\text{know}}]{𝐖}_{\text{fc}}+{𝐛}_{\text{fc}}\right)\text{,} \end{array}$$ (6)

where [·∥·] denotes the concatenation operation, and ${𝐖}_{\text{fc}},{𝐛}_{\text{fc}}$ are the parameters of the fusion layer.

Finally, the network parameters Θ are learned by minimizing the loss function ℒ between the predicted proportions $\hat{y}$ and the ground truth 𝐲 across the training set:


$$\begin{array}{c} \Theta^{*}=\text{arg}\min_{\Theta }\sum_{(𝐱,𝐲)\in 𝒟}ℒ(f_{\Theta }(𝐱),𝐲)\text{.} \end{array}$$ (7)

#### Simulation of pseudo-bulk data from scRNA-seq data.

Since supervised training of the iDCF model requires a large number of samples with known cell proportions, whereas real bulk data often lacks such annotations, we employ a strategy of simulating pseudo-bulk samples from scRNA-seq data to construct the training set. This simulation process, which is based on single-cell expression profiles with accurate cell-type annotations, proceeds as follows:

First, for each simulated sample, a target cell-type proportion vector $\theta =[\theta_{1},\theta_{2},...,\theta_{C}]$ was generated, subject to the unit simplex constraint $\sum_{c=1}^{C}\theta_{c}=1$, where *C* denotes the total number of cell types. We provide two probabilistic models for generating this vector. In scenarios incorporating prior knowledge or specific sparsity assumptions, proportions were sampled from a Dirichlet distribution $\theta \sim \text{Dir}(\alpha_{1},\alpha_{2},...,\alpha_{C})$, where the concentration parameters control both the expected abundance and dispersion; a symmetric Dirichlet distribution ($\alpha_{c}=1,\forall c\in 1,...,C$) was typically employed in the absence of informative priors. Alternatively, to simulate random compositions without distributional assumptions, unnormalized weights were sampled independently from a standard uniform distribution $u_{c}\sim U(0,1)$ and subsequently normalized according to $\theta_{c}=u_{c}/\sum_{j=1}^{C}u_{j}$.

To mimic the biological reality where specific cell types may be absent in certain samples, a sparsity-inducing mechanism was introduced. For a subset of simulated samples, specific cell types were randomly selected and their proportions set to zero, followed by the renormalization of the remaining non-zero fractions to unity.

To capture the stochasticity inherent in cell dissociation and library preparation, the final cell counts were modeled using a Poisson distribution, a standard approach for approximating technical variance in sequencing reads [71]. First, the expected cell count $\lambda_{c}$ for cell type *c* was calculated as $\lambda_{c}=N_{total}\times \theta_{c}$, where $N_{total}$ denotes the predefined total library size and θ is the proportion vector derived above. The actual abundance $N_{c}$ was then drawn from:


$$\begin{array}{c} N_{c}\sim \text{Poisson}(\lambda_{c})\text{.} \end{array}$$ (8)

To prevent biologically implausible outliers, $N_{c}$ values were constrained within a predefined fluctuation range around the expectation.

Based on the determined count $N_{c}$, single-cell expression profiles were sampled with replacement from the corresponding cell-type specific pool. The final gene expression vector ${𝐱}_{p}$ for a pseudo-bulk sample was constructed by aggregating the transcript counts of all recruited cells:


$$\begin{array}{c} x_{p,g}=\sum_{c=1}^{C}\sum_{i=1}^{N_{c}}E_{g,c,i}\text{,} \end{array}$$ (9)

where $x_{p,g}$ represents the accumulated expression of gene *g* in the pseudo-bulk sample, and $E_{g,c,i}$ denotes the expression of gene *g* in the *i*-th single-cell profile sampled for cell type *c*.

To further enhance simulation fidelity, an optional noise-injection module was implemented. A Gaussian noise term $\epsilon \sim 𝒩(0,\sigma^{2})$ was superimposed onto the final aggregated profiles to emulate residual technical noise arising from the sequencing process. This comprehensive pipeline was utilized to generate a diverse training cohort of 8,000 pseudo-bulk samples for each reference dataset.

#### Input data preprocessing.

To ensure robust model generalization from pseudo-bulk training data to real bulk target data, a standardized preprocessing pipeline was implemented. First, to construct a unified feature space for training and inference, low-variance filtering was applied independently to the training and test datasets to exclude uninformative features. The final feature set was defined as the intersection of genes retained in both datasets, ensuring consistent input dimensions across domains.

Subsequently, the aligned expression matrices underwent a two-step normalization process. All raw expression values were first log-transformed using $x=\log_{2}(x+1)$ to stabilize variance. To preserve the relative ranking of gene expression within each profile, sample-wise Min-Max scaling was applied. For a given sample vector ${𝐱}_{i}$, the normalized expression value ${\tilde{x}}_{i,j}$ was linearly rescaled to the [0,1] interval:


$$\begin{array}{c} {\tilde{x}}_{i,j}=\frac{x_{i,j}-\min({𝐱}_{i})}{\max({𝐱}_{i})-\min(𝐱i)}\text{,} \end{array}$$ (10)

where $x_{i,j}$ denotes the log-transformed expression level of gene *j* in sample *i*, and $\min({𝐱}_{i})$ and $\max({𝐱}_{i})$ represent the minimum and maximum expression values within that sample, respectively.

#### Architecture and training procedure.

The iDCF framework features a dual-stream neural architecture designed to process transcriptomic data through complementary mechanisms. The data-driven stream (DNN) comprises a sequence of five fully connected layers with diminishing dimensionality (512, 256, 128, 64, and 32 neurons, respectively). In parallel, the knowledge-guided stream (KSNN) initiates with a biologically constrained sparse layer, where the number of neurons corresponds to the total number of knowledge features, followed by five fully connected layers mirroring the structure of the DNN. Comprehensive architectural diagrams and hyperparameter details are provided in S5 Fig.

The structural constraints of the KSNN are defined by a knowledge mask matrix, constructed by integrating biological pathway annotations and PPI networks. For pathway information, gene sets were retrieved from the Molecular Signatures Database (MSigDB) [72,73], encompassing the KEGG, Reactome, PID, and BioCarta repositories. From an initial pool of 2,410 pathways, a filtering strategy was applied to enhance specificity: pathways were retained if they contained between 15 and 300 genes, or if they exceeded 15 genes while maintaining an overlap rate of strictly less than 1 with other pathways to ensure distinctness. This process yielded 1,603 high-quality pathways, which were encoded into a binary gene-by-pathway matrix.

Simultaneously, PPI data were sourced from the STRING database [15]. To ensure reliability, interactions were rigorously filtered to retain only those with a high confidence score (>700). An adjacency matrix was constructed for genes present in the final feature space, initialized with identity diagonal elements to represent self-associations. Off-diagonal entries for interacting gene pairs (*i*, *j*) were populated with weighted values derived from the normalized STRING confidence score (score divided by 1,000). Finally, the binary pathway matrix and the weighted PPI adjacency matrix were concatenated along the feature dimension to constitute the final biological knowledge mask matrix used to initialize and constrain the KSNN.

The iDCF model was implemented using the PyTorch framework. Network parameters were optimized using the Adam algorithm with a fixed learning rate of $1\times 10^{-4}$. Training was conducted for 128 epochs with a batch size of 64 using the pseudo-bulk cohorts described above. To balance sensitivity to outliers with convergence stability, a composite objective function combining mean squared error (MSE) and mean absolute error (MAE) was minimized:


$$\begin{array}{c} ℒ(\hat{y},𝐲)={ℒ}_{\text{MSE}}+{ℒ}_{\text{MAE}}=\frac{1}{c}\sum_{j=1}^{c}({\hat{y}}_{j}-y_{j}{)}^{2}+\frac{1}{c}\sum_{j=1}^{c}|{\hat{y}}_{j}-y_{j}|\text{,} \end{array}$$ (11)

where *c* denotes the total number of cell types.

To mitigate stochastic fluctuations arising from random weight initialization and enhance predictive robustness, an ensemble strategy was adopted. Three independent models were trained in parallel, and the final proportion estimate for cell type *c*, denoted as ${\hat{y}}_{c}$, was derived by averaging the outputs of the individual models:


$$\begin{array}{c} {\hat{y}}_{c}=\frac{1}{3}\sum_{i=1}^{3}{\hat{y}}_{c}^{i}\text{,} \end{array}$$ (12)

where $\hat{y}c^{\left(i\right)}$ represents the prediction from the *i*-th independent model instance.

#### SHAP interpretability module.

To decipher the internal decision-making logic of iDCF, the SHAP framework [20] was integrated to quantify feature attribution. The GradientExplainer algorithm, optimized for deep neural networks, was employed to approximate Shapley values. A background reference set comprising 1,000 randomly sampled training instances was utilized to estimate expected model values; for the specific case of sEV functional analysis, the background consisted of TCGA CRC tumor and matched normal samples. Interpretability analysis was conducted at two granularities (Fig 1B):

To identify molecular drivers of specific cell types, SHAP values were computed for individual genes relative to the final predicted proportions. This analysis preserved directional information, allowing for the determination of whether specific gene expression levels exerted a positive or negative influence on the estimation of a given cell type. This granular attribution enabled the identification of key marker genes governing cell identity.

To interrogate the biological priors encoded by the KSNN, SHAP values were also computed for individual knowledge features (pathways and PPI nodes) relative to the predicted cell-type proportions. As with the gene-level analysis, these values determine whether the activation of a specific biological module exerts a positive or negative influence on a given cell type.

### Performance evaluation

To quantitatively assess the predictive performance of iDCF against state-of-the-art benchmarks, two core metrics were employed: mean absolute error (MAE) and Lin’s concordance correlation coefficient (CCC). MAE quantifies the average magnitude of the deviation between predicted and ground truth cell proportions. It is calculated as:


$$\begin{array}{c} \text{MAE}=\frac{1}{n}\sum_{i=1}^{n}|y_{i}-{\hat{y}}_{i}|\text{,} \end{array}$$ (13)

where *n* denotes the total number of observations, and $y_{i}$ and ${\hat{y}}_{i}$ represent the ground truth and predicted proportions for the *i*-th data point, respectively.

CCC provides a comprehensive measure of agreement that accounts for both the correlation and the deviation from the identity line (precision and accuracy). It is defined as:


$$\begin{array}{c} \text{CCC}=\frac{2r\sigma_{y}\sigma_{\hat{y}}}{\sigma_{y}^{2}+\sigma_{\hat{y}}^{2}+(\mu_{y}-\mu_{\hat{y}}{)}^{2}}\text{,} \end{array}$$ (14)

where $\mu_{y}$ and $\mu_{\hat{y}}$ are the means of the true and predicted values, $\sigma_{y}^{2}$ and $\sigma_{\hat{y}}^{2}$ are their respective variances, and *r* is the Pearson correlation coefficient between 𝐲 and $\hat{y}$.

### Algorithm comparison

To evaluate its performance, iDCF was benchmarked against several state-of-the-art methods, including DeSide, scpDeconv, TAPE, Scaden, ReDeconv, BayesPrism, and CIBERSORTx. To ensure a fair comparison, all methods were evaluated using identical single-cell reference data.

#### DeSide.

DeSide [14] is a deep learning deconvolution method based on DNN, which employs two parallel fully connected neural networks to extract features from gene expression data and pathway data, respectively. DeSide is primarily designed for analyzing solid tumors, but it is also applicable to non-tumor settings. When analyzing solid tumors, it adopts Sigmoid as the activation function of the output layer, predicting the proportion of non-cancer cells with soft constraints, and then deriving the cancer cell proportion by subtraction. In this study, we adopted the mode suitable for non-tumor data, specifically, using Softmax as the activation function of the output layer to directly predict the proportions of different cell types. We trained the model using the source code provided by the authors and followed their official guidelines.

#### scpDeconv.

scpDeconv [21] is a deep learning method that integrates hybrid strategies, autoencoders, and domain adversarial networks. It employs an autoencoder to enhance the quality of the single-cell reference data using information from bulk data, and a domain adversarial architecture to bridge the distributions between single-cell and bulk data, thereby transferring label information from the single-cell domain to the bulk domain. Given that the reference data in this study were predefined, we omitted its first stage of reference data preparation and directly applied the source code provided by the authors for the subsequent analysis.

#### TAPE.

TAPE [13] is an autoencoder-based deep learning method comprising three workflow stages: pseudo-bulk simulation, model training, and sample adaptation. The framework offers two operating modes: Overall and High-resolution. In Overall mode, TAPE processes the entire cohort simultaneously to generate a universal signature matrix; in High-resolution mode, it processes samples individually to construct sample-specific adjusted signature matrices. We evaluated both configurations, hereafter referred to as TAPE-O (Overall) and TAPE-H (High-resolution). Analyses were conducted using the official scTAPE Python package (v1.1.2) following the standard workflow.

#### Scaden.

Scaden [12] is a deep learning deconvolution method utilizing a deep neural network (DNN) architecture with four fully connected layers. The final predictive model employs an ensemble strategy, averaging the outputs of the three top-performing independently trained models. We performed training and inference using the official Scaden package (v1.1.2) according to the recommended guidelines.

#### ReDeconv.

ReDeconv [22] integrates effective transcriptome size into scRNA-seq normalization and bulk deconvolution. It applies a strategy termed CTLS (Counts based on linearized transcriptome size) to correct for biases in differential expression analysis that may arise from standard normalization methods (e.g., counts per 10K). Furthermore, ReDeconv enhances performance by explicitly modeling expression heterogeneity and reducing gene length effects. We implemented this method using the official software provided by the authors (https://redeconv.stjude.org/software).

#### BayesPrism.

BayesPrism [10] is a Bayesian framework designed for cancer tissue deconvolution. It constructs a prior model from cell-type-specific gene expression profiles derived from scRNA-seq to jointly infer cell type composition and the posterior distribution of gene expression in bulk RNA-seq data. Analyses were performed using the official R package (v2.2.2) via the standard pipeline.

#### CIBERSORTx.

CIBERSORTx [9] is a support vector regression (SVR)-based method capable of leveraging single-cell reference profiles for deconvolution. We performed the analysis using the official web platform (https://cibersortx.stanford.edu/). Specifically, a signature matrix was first constructed from the single-cell reference data; subsequently, S-mode batch correction was applied to mitigate cross-platform variation while estimating cell proportions. All other parameters were maintained at their default values.

## Discussion

Computational cell deconvolution serves as a critical bridge for unlocking cellular insights from the vast archives of legacy bulk transcriptomic data. However, the field has long faced a fundamental accuracy-interpretability dilemma. In this work, we introduced iDCF, a framework that resolves this trade-off by explicitly embedding biological priors into the deep learning architecture. Through rigorous benchmarking across diverse tissue contexts, from PBMCs to complex tumor microenvironments and neurodegenerative lesions, we demonstrated that iDCF establishes a new state-of-the-art in predictive accuracy while providing the mechanistic transparency requisite for clinical adoption.

The core innovation of iDCF lies in its use of a biologically informed network architecture. Unlike conventional data-driven models that treat gene expression as unstructured vectors, iDCF leverages the KSNN to impose biological topology directly onto the neural connectivity. By using knowledge masks to define the network structure, the model restricts information flow to biologically valid pathways and PPI modules. Our ablation studies confirm that this design does not simply accumulate redundant data; instead, the integration of pathway and PPI complementary constraints that are critical for model robustness. This approach ensures that the model learns features that are not only statistically predictive but also biologically meaningful.

In performance evaluations, iDCF exhibited exceptional resolution in distinguishing highly homologous lymphocyte subsets. It consistently outperformed advanced baselines in resolving B cells, CD4 T cells, and CD8 T cells. Regarding monocytes, we observed that iDCF’s advantage was less pronounced compared to methods like CIBERSORTx. Crucially, the interpretability module of iDCF provided a diagnostic explanation for this observation. SHAP analysis (S4 Fig) revealed that while the model correctly identified key markers such as TLR4, its decision boundary was partly confounded by non-specific signals like CYP1B1. This transparency suggests that the performance bottleneck for monocytes stems from a low signal-to-noise ratio in the specific training features rather than a fundamental flaw in the model’s logic. Unlike opaque “black-box” models that fail without explanation, iDCF offers the transparency to identify why a prediction may be suboptimal, enabling targeted refinement of future reference atlases.

A defining feature of iDCF is its capacity to demystify the “black box” of deep learning through mechanistic transparency. By integrating the SHAP framework, we successfully dissected the molecular logic governing the model’s inference. Using B cells as a paradigm, our analysis revealed a fundamental shift in feature attribution: in the absence of knowledge guidance, the baseline model relied on spurious, unrelated genes, resulting in decision patterns driven by stochastic noise. In contrast, the knowledge-integrated iDCF model correctly locked onto canonical markers and functional hubs such as CD19, MS4A1, and PAX5, establishing a robust and biologically intuitive decision boundary. Crucially, KEGG enrichment analysis of the genes with the highest feature weights confirms that the model is not merely selecting isolated marker genes, but is leveraging coherent gene modules. This finding validates that the KSNN is operating as designed, processing information through functional units rather than disparate loci. This transparency extends the utility of deconvolution beyond simple cellular quantification to the discovery of functional states. We exemplified this in our analysis of colorectal cancer sEVs. By comparing the top-contributing transcriptomic features in normal versus malignant tissues, iDCF’s interpretability framework uncovered a functional reprogramming of the vesicular secretome: shifting from a homeostatic role in tissue maintenance to a pro-tumorigenic phenotype driving progression. This demonstrates that iDCF can serve as a hypothesis-generation tool for uncovering cryptic biological mechanisms within the tumor microenvironment.

To validate translational utility, we benchmarked iDCF across a spectrum of heterogeneous clinical landscapes. In the context of neurodegeneration, iDCF went beyond static counting to accurately reconstruct temporal pathologies, capturing both the progressive loss of neurons and the complex, non-linear dynamics of microglia associated with AD staging. Extending this precision to solid tumor oncology, the model faithfully resolved the immune architecture of the HGSC microenvironment, reproducing infiltration patterns of macrophages and CD8 T cells consistent with multiplex immunofluorescence validation. Furthermore, in metabolic disease, iDCF demonstrated high sensitivity to physiological dysregulation; the predicted beta-cell mass exhibited the strongest negative correlation with HbA1c levels, effectively serving as a digital biomarker for the pathophysiological state of T2D. Collectively, these diverse applications provide compelling evidence that iDCF is a robust and versatile tool, capable of extracting reliable biological insights from complex, multi-tissue transcriptomic data.

We acknowledge certain limitations inherent to our knowledge-driven paradigm. First, the performance of iDCF is inextricably linked to the fidelity of the single-cell reference and the completeness of the biological priors. For understudied tissues or rare cell states where pathway annotations are sparse, constructing effective knowledge masks remains a challenge. Second, our reliance on static knowledge bases represents a current limitation. Cellular interactions are context-dependent and plastic, particularly in disease states. Future iterations of iDCF could incorporate dynamic masking mechanisms, where network topology is inferred or adjusted via multi-modal integration (e.g., ATAC-seq or spatial transcriptomics), allowing the model to adapt to the rewired interactomes characteristic of pathology.

In summary, iDCF represents a methodological convergence between deep learning and systems biology. By explicitly encoding biological laws into neural networks, we overcome the inherent opacity of state-of-the-art deep learning methods. iDCF stands not merely as a tool for estimating cell proportions, but as a transparent analytical framework that validates the biological soundness of its own decisions. We anticipate that iDCF will serve as a powerful instrument for dissecting cellular heterogeneity in complex tissues, empowering the community to extract robust, interpretable insights from the vast landscape of bulk transcriptomic data.

## Supporting information

S1 Fig Performance evaluation of iDCF and benchmark methods on different bulk datasets. (A) Performance comparison of iDCF and seven benchmark methods on eight independent PBMC bulk datasets. Box plots show the distribution of mean absolute error (MAE) between predicted cell-type proportions and flow cytometry ground truth. Lower MAE values indicate smaller prediction errors and higher accuracy. The Lin’s concordance correlation coefficient (CCC) comparison is presented in Fig 3A of the main text. (B, C) Performance comparison of iDCF and seven benchmark methods on the ROSMAP human brain bulk dataset, showing results using (B) CCC and (C) MAE as evaluation metrics. In the box plots, the center line represents the median, the box edges represent the upper and lower quartiles, and the whiskers indicate 1.5 times the interquartile range.
(PDF)

S2 Fig Prediction of beta cell fractions in SARS-CoV-2 infected islet samples by iDCF and benchmark methods. (A) Predicted beta cell fractions for two independent donors (left: Sample S_2, right: Sample S_3) from the GSE159717 dataset. Results are shown across three conditions: uninfected (Control), infected with SARS-CoV-2 (SARS-CoV-2), and post-infection treatment with remdesivir (SARS-CoV-2 + Remdesivir). This panel evaluates the ability of each method to capture the decline in beta cell proportion caused by viral infection and the recovery trend following drug treatment. (B) Predicted beta cell fractions for an independent validation cohort (GSE165890) comprising 12 primary human pancreatic islet samples (6 healthy controls, 6 SARS-CoV-2 infected). Bar heights represent mean predicted fractions, with error bars indicating standard deviation. Statistical significance was assessed using a one-tailed t-test based on the established biomedical consensus that SARS-CoV-2 infection specifically destroys pancreatic beta cells. iDCF, DeSide, TAPE, and ReDeconv detected a significant depletion (** *p* < 0.01), Scaden and BayesPrism showed weak significance (* *p* < 0.05), while scpDeconv and CIBERSORTx did not reveal a statistically significant difference (ns). This independent validation provides quantitative summary statistics supporting iDCF’s reliable performance ranking.
(PDF)

S3 Fig Interpretability analysis of iDCF reveals functional shifts of small extracellular vesicles (sEVs) in colorectal cancer. (A, B) SHAP value summary plots of the top 20 biological knowledge features (pathways or PPI genes) contributing most to model predictions in cancer samples (A) and adjacent normal tissue samples (B). (C, D) SHAP value summary plots of the top 20 genes contributing most to model predictions in cancer samples (C) and adjacent normal tissue samples (D).
(PDF)

S4 Fig Interpretability analysis of the influence of biological knowledge on iDCF decision-making. SHAP summary plots show the top 20 genes contributing most to predictions of different cell types in the Monaco 1 dataset, with or without biological knowledge guidance. Each point represents a sample, with color indicating gene expression level (red = high, blue = low) and position on the x-axis denoting the SHAP value of that gene for the prediction outcome. (A–E) Top 20 genes relied upon by the model to predict the fractions of CD4 T cells (A), CD8 T cells (B), monocytes (C), NK cells (D), and unknown cells (E) without biological knowledge guidance. (F–J) Top 20 genes relied upon by the model to predict the fractions of CD4 T cells (F), CD8 T cells (G), monocytes (H), NK cells (I), and unknown cells (J) after integrating biological knowledge.
(PDF)

S5 Fig Detailed network architectures of the KSNN and DNN modules in the iDCF model. (A) Architecture of the knowledge-based sparse neural network (KSNN). After inputting gene expression profiles, gene features are mapped to a knowledge feature space through a sparse connection layer guided by biological knowledge (knowledge mask). The resulting feature vectors then pass through five fully connected (Linear) modules, each consisting of a linear layer and a ReLU activation function. The last three modules also include a LayerNorm layer. (B) Architecture of the deep neural network (DNN). The network consists of five fully connected modules, each containing a linear layer, a ReLU activation function, and a Dropout layer (with a dropout rate of 0.05 for the first three layers and 0.2 for the fourth layer; the final module excludes Dropout). Some modules are also followed by a LayerNorm layer.
(PDF)

S6 Fig Heatmap summary of deconvolution performance across cell types and methods. Pearson correlation coefficients (*r*) between predicted and ground truth cell proportions are shown for each cell type and each method. (A) Performance on the PBMC6k pseudo-bulk test dataset. (B) Performance on the DonorA pseudo-bulk test dataset. Colors range from blue (low correlation) to red (high correlation), as indicated by the color bar. iDCF consistently achieves the highest or near-highest correlation for most cell types across both datasets.
(PDF)

S1 Table Summary of the single-cell RNA sequencing (scRNA-seq) reference dataset used in this study. (PDF)

S2 Table Relationship between CellTypist annotated cell types and major cell types. (PDF)

S3 Table Summary of the bulk RNA sequencing (RNA-seq) dataset used for deconvolution evaluation. (PDF)

S4 Table Relationship between cell types in bulk datasets and major cell types. (PDF)

S5 Table Performance comparison (Pearson correlation coefficient *r*) of different deconvolution methods across PBMC6k and DonorA datasets. (PDF)

S6 Table Evaluation of deconvolution performance based on CCC and MAE across different cell types. (PDF)

S7 Table Statistical comparison of deconvolution performance between Control and SARS-CoV-2 groups across different methods. (PDF)

S1 Data Source data for all figures in the main text and supplementary information. (ZIP)
