# Supplementary material for: iDCF: Interpretable deconvolution of cell fractions via biologically-informed deep learning using scRNA-seq data
Source: PLoS Comput Biol. 2026 Aug 27;22(8):e1014727. doi: 10.1371/journal.pcbi.1014727 (PMC13552964; doi:10.1371/journal.pcbi.1014727)
Supplement: Supplementary file 1 [file pcbi.1014727.s001.pdf]

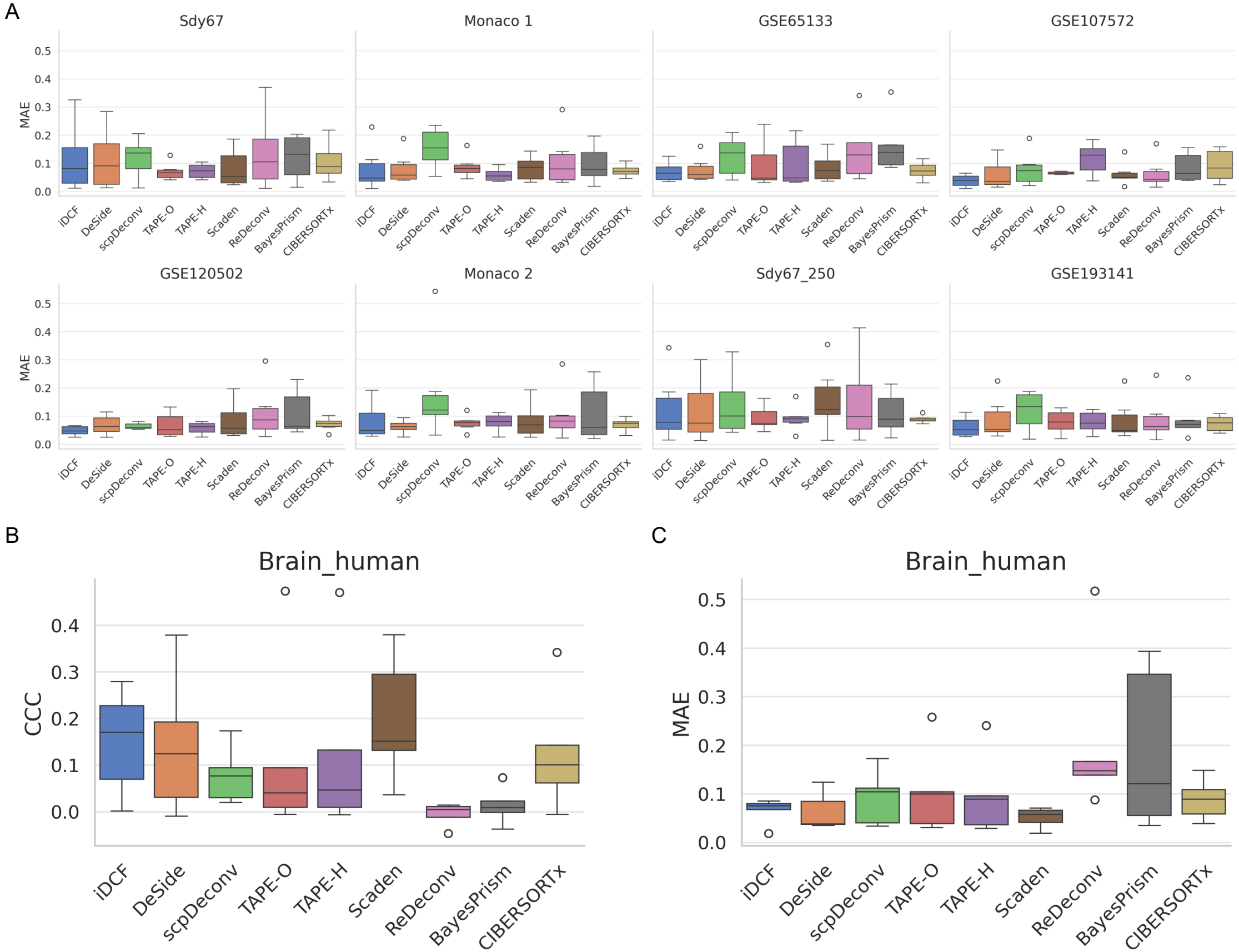

S1 Fig. Performance evaluation of iDCF and benchmark methods on different bulk datasets. (A) Performance comparison of iDCF and seven benchmark methods on eight independent PBMC bulk datasets. Box plots show the distribution of mean absolute error (MAE) between predicted cell-type proportions and flow cytometry ground truth. Lower MAE values indicate smaller prediction errors and higher accuracy. The Lin's concordance correlation coefficient (CCC) comparison is presented in Fig 3A of the main text. (B, C) Performance comparison of iDCF and seven benchmark methods on the ROSMAP human brain bulk dataset, showing results using (B) CCC and (C) MAE as evaluation metrics. In the box plots, the center line represents the median, the box edges represent the upper and lower quartiles, and the whiskers indicate 1.5 times the interquartile range.
