# Supplementary material for: iDCF: Interpretable deconvolution of cell fractions via biologically-informed deep learning using scRNA-seq data
Source: PLoS Comput Biol. 2026 Aug 27;22(8):e1014727. doi: 10.1371/journal.pcbi.1014727 (PMC13552964; doi:10.1371/journal.pcbi.1014727)
Supplement: Supplementary file 2 [file pcbi.1014727.s002.pdf]

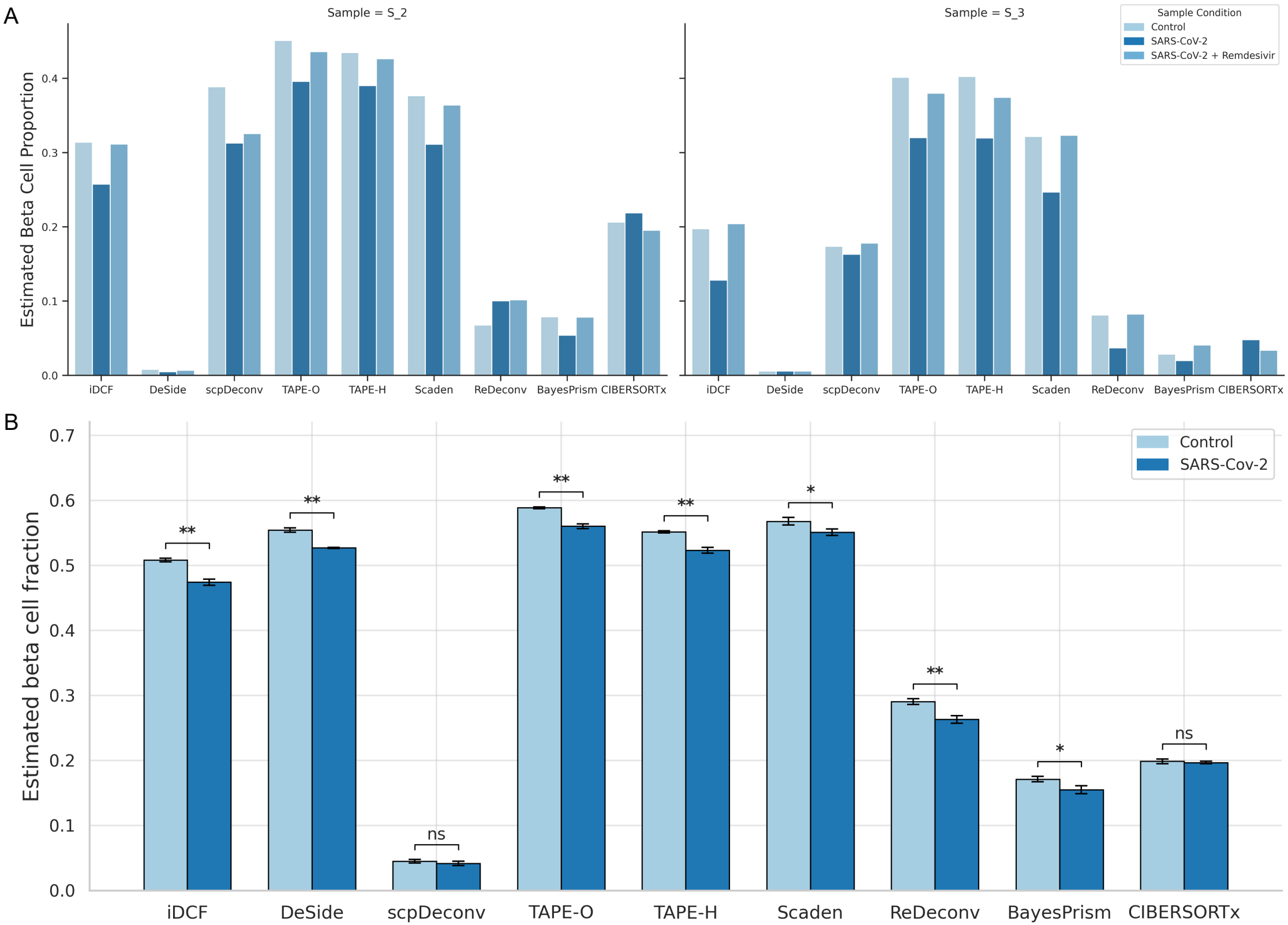

S2 Fig. Prediction of beta cell fractions in SARS-CoV-2 infected islet samples by iDCF and benchmark methods. (A) Predicted beta cell fractions for two independent donors (left: Sample S_2, right: Sample S_3) from the GSE159717 dataset. Results are shown across three conditions: uninfected (Control), infected with SARS-CoV-2 (SARS-CoV-2), and post-infection treatment with remdesivir (SARS-CoV-2 + Remdesivir). This panel evaluates the ability of each method to capture the decline in beta cell proportion caused by viral infection and the recovery trend following drug treatment. (B) Predicted beta cell fractions for an independent validation cohort (GSE165890) comprising 12 primary human pancreatic islet samples (6 healthy controls, 6 SARS-CoV-2 infected). Bar heights represent mean predicted fractions, with error bars indicating standard deviation. Statistical significance was assessed using a one-tailed t-test based on the established biomedical consensus that SARS-CoV-2 infection specifically destroys pancreatic beta cells. iDCF, DeSide, TAPE, and ReDeconv detected a significant depletion (**: $p < 0.01$), Scaden and BayesPrism showed weak significance (*: $p < 0.05$), while scpDeconv and CIBERSORTx did not reveal a statistically significant difference (ns). This independent validation provides quantitative summary statistics supporting iDCF's reliable performance ranking.
