# Supplementary material for: iDCF: Interpretable deconvolution of cell fractions via biologically-informed deep learning using scRNA-seq data
Source: PLoS Comput Biol. 2026 Aug 27;22(8):e1014727. doi: 10.1371/journal.pcbi.1014727 (PMC13552964; doi:10.1371/journal.pcbi.1014727)
Supplement: Supplementary file 3 [file pcbi.1014727.s003.pdf]

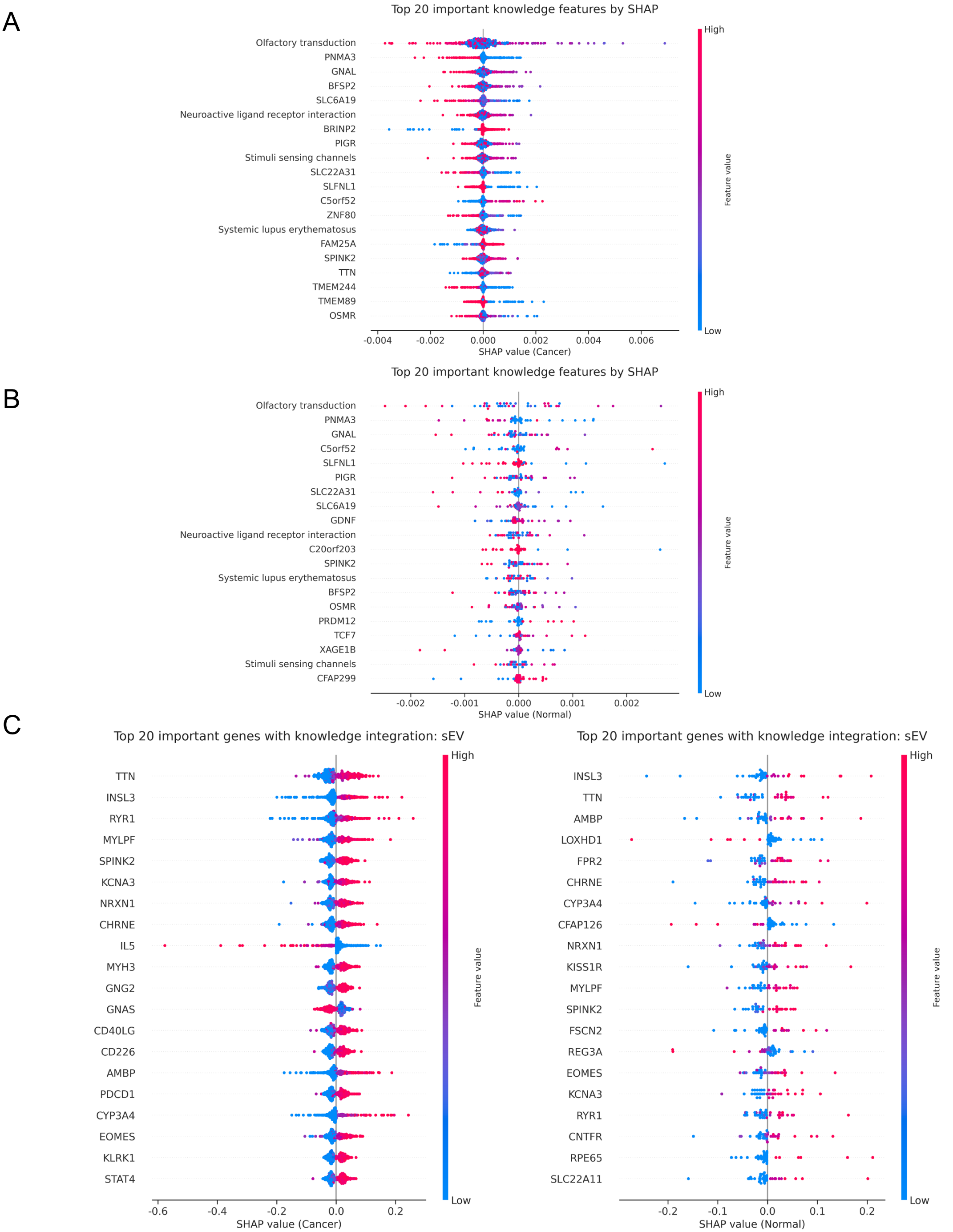

S3 Fig. Interpretability analysis of iDCF reveals functional shifts of small extracellular vesicles (sEVs) in colorectal cancer. (A, B) SHAP value summary plots of the top 20 biological knowledge features (pathways or PPI genes) contributing most to model predictions in cancer samples (A) and adjacent normal tissue samples (B). (C, D) SHAP value summary plots of the top 20 genes contributing most to model predictions in cancer samples (C) and adjacent normal tissue samples (D).
