# Supplementary material for: iDCF: Interpretable deconvolution of cell fractions via biologically-informed deep learning using scRNA-seq data
Source: PLoS Comput Biol. 2026 Aug 27;22(8):e1014727. doi: 10.1371/journal.pcbi.1014727 (PMC13552964; doi:10.1371/journal.pcbi.1014727)
Supplement: Supplementary file 4 [file pcbi.1014727.s004.pdf]

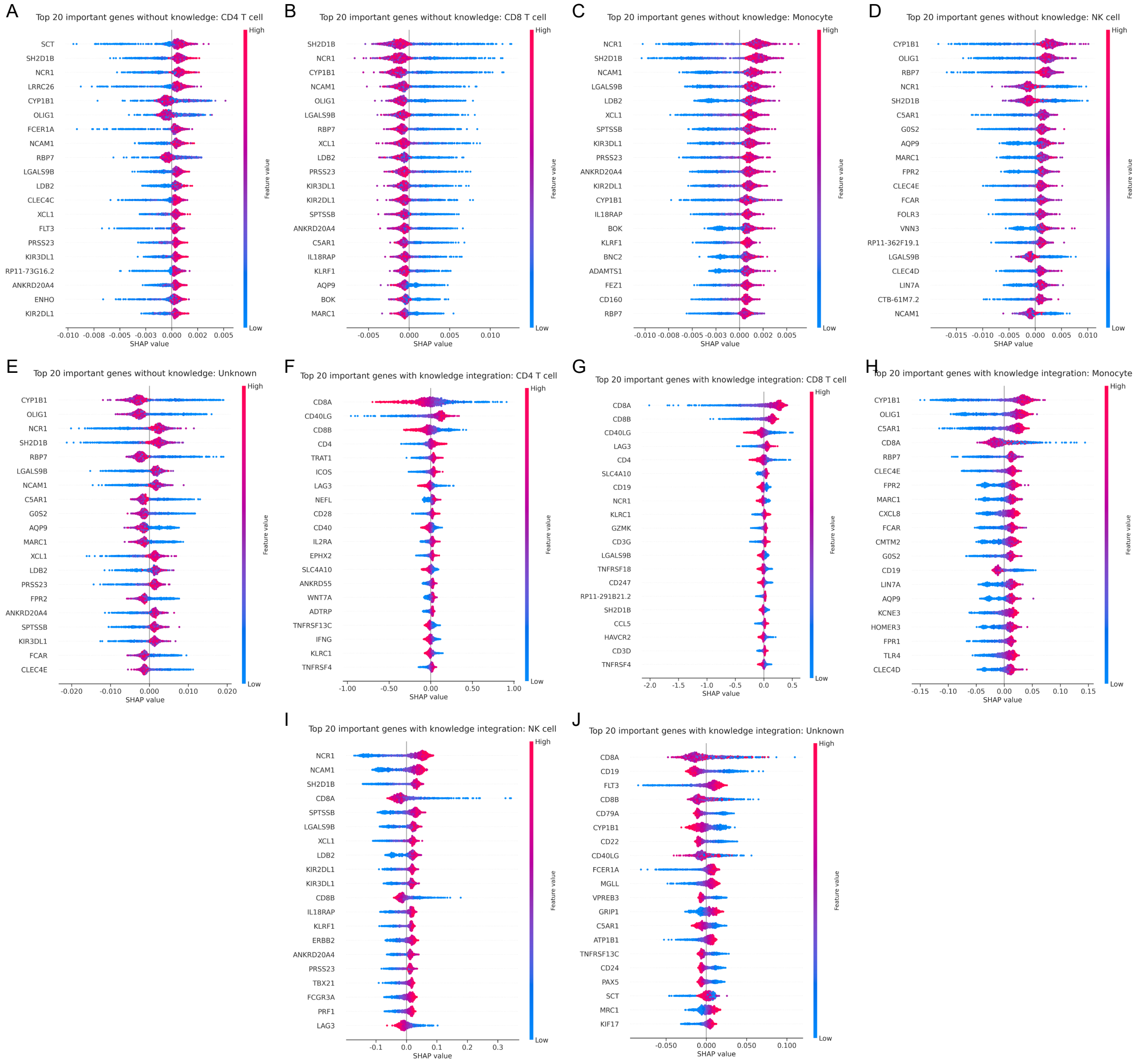

S4 Fig. Interpretability analysis of the influence of biological knowledge on iDCF decision-making. SHAP summary plots show the top 20 genes contributing most to predictions of different cell types in the Monaco 1 dataset, with or without biological knowledge guidance. Each point represents a sample, with color indicating gene expression level (red = high, blue = low) and position on the x-axis denoting the SHAP value of that gene for the prediction outcome. (A–E) Top 20 genes relied upon by the model to predict the fractions of CD4 T cells (A), CD8 T cells (B), monocytes (C), NK cells (D), and unknown cells (E) without biological knowledge guidance. (F–J) Top 20 genes relied upon by the model to predict the fractions of CD4 T cells (F), CD8 T cells (G), monocytes (H), NK cells (I), and unknown cells (J) after integrating biological knowledge.
