# Supplementary material for: iDCF: Interpretable deconvolution of cell fractions via biologically-informed deep learning using scRNA-seq data
Source: PLoS Comput Biol. 2026 Aug 27;22(8):e1014727. doi: 10.1371/journal.pcbi.1014727 (PMC13552964; doi:10.1371/journal.pcbi.1014727)
Supplement: Supplementary file 5 [file pcbi.1014727.s005.pdf]

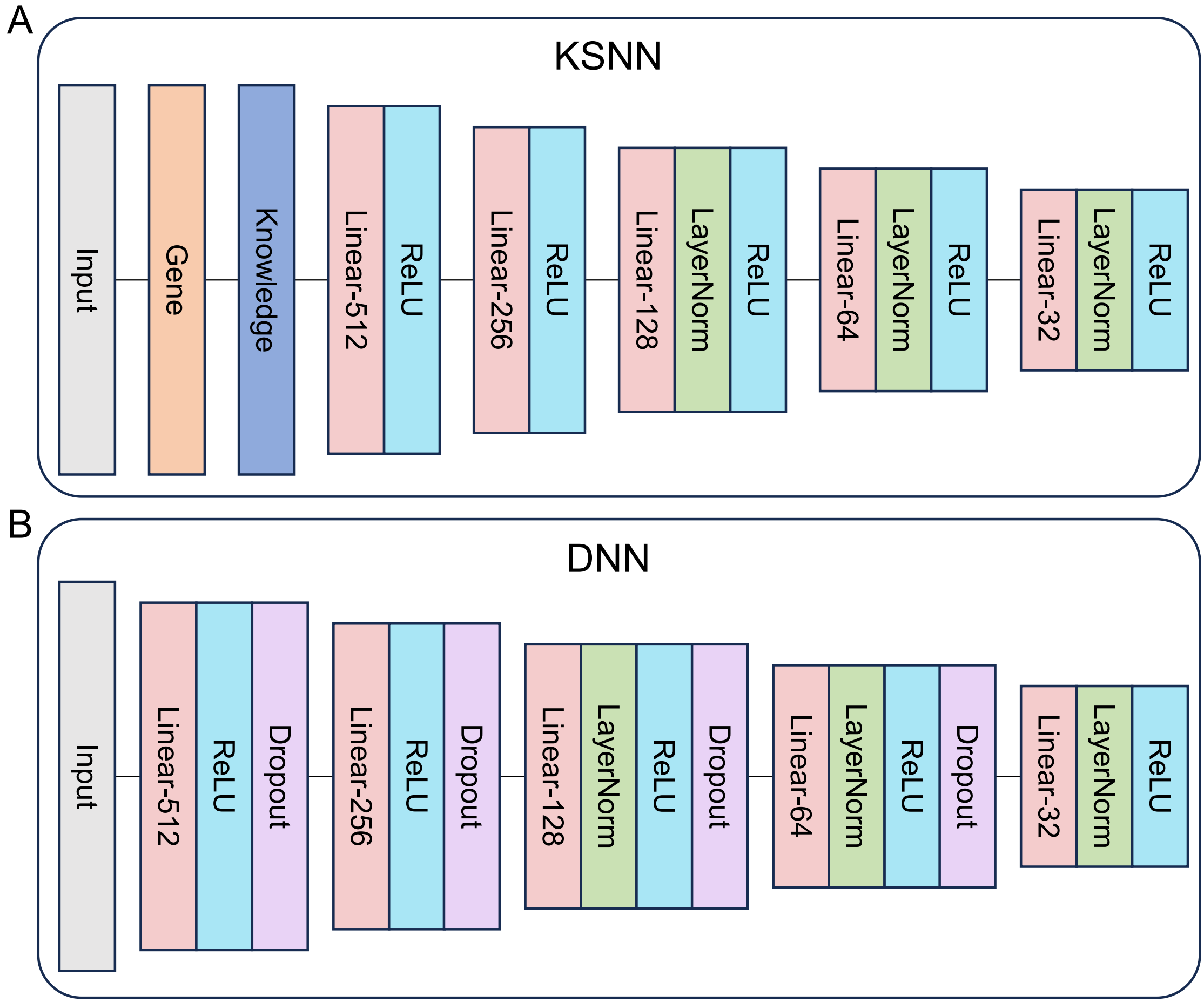

S5 Fig. Detailed network architectures of the KSNN and DNN modules in the iDCF model. (A) Architecture of the knowledge-based sparse neural network (KSNN). After inputting gene expression profiles, gene features are mapped to a knowledge feature space through a sparse connection layer guided by biological knowledge (knowledge mask). The resulting feature vectors then pass through five fully connected (Linear) modules, each consisting of a linear layer and a ReLU activation function. The last three modules also include a LayerNorm layer. (B) Architecture of the deep neural network (DNN). The network consists of five fully connected modules, each containing a linear layer, a ReLU activation function, and a Dropout layer (with a dropout rate of 0.05 for the first three layers and 0.2 for the fourth layer; the final module excludes Dropout). Some modules are also followed by a LayerNorm layer.
