# Supplementary material for: iDCF: Interpretable deconvolution of cell fractions via biologically-informed deep learning using scRNA-seq data
Source: PLoS Comput Biol. 2026 Aug 27;22(8):e1014727. doi: 10.1371/journal.pcbi.1014727 (PMC13552964; doi:10.1371/journal.pcbi.1014727)
Supplement: Supplementary file 6 [file pcbi.1014727.s006.pdf]

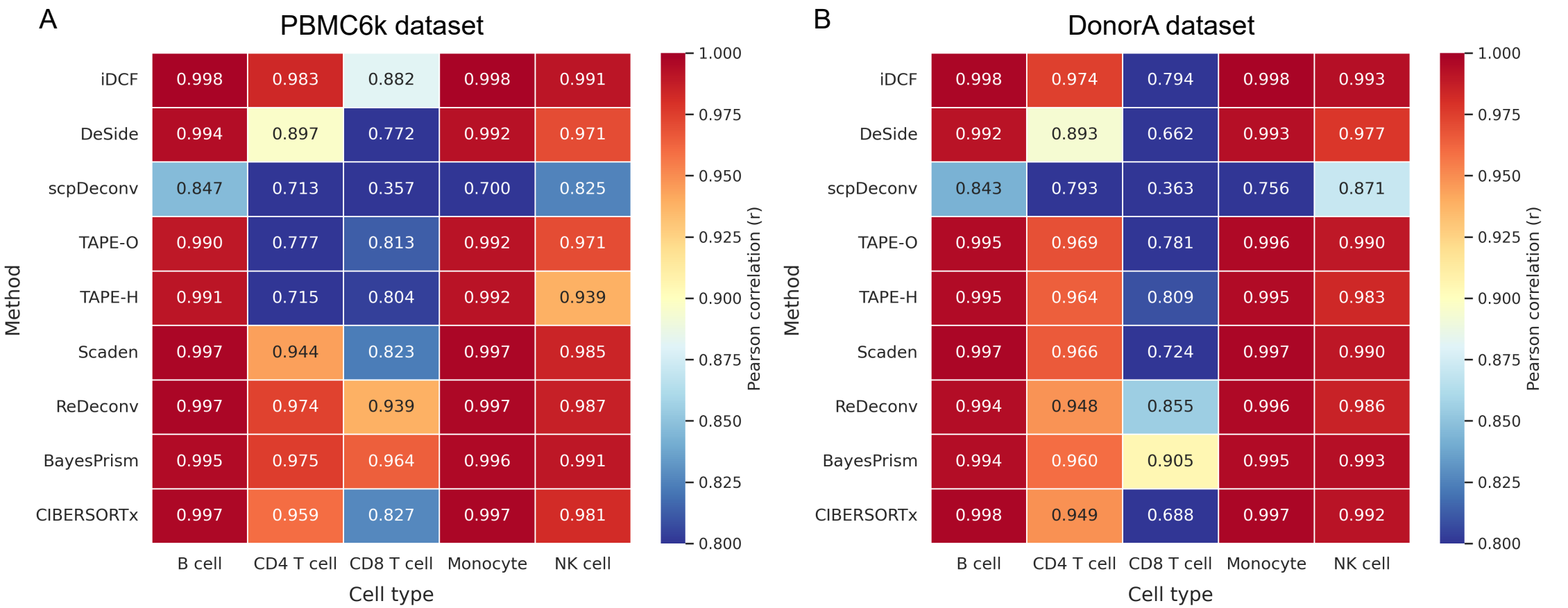

S6 Fig. Heatmap summary of deconvolution performance across cell types and methods. Pearson correlation coefficients (r) between predicted and ground truth cell proportions are shown for each cell type and each method. (A) Performance on the PBMC6k pseudo-bulk test dataset. (B) Performance on the DonorA pseudo-bulk test dataset. Colors range from blue (low correlation) to red (high correlation), as indicated by the color bar. iDCF consistently achieves the highest or near-highest correlation for most cell types across both datasets.
