# Supplementary material for: iDCF: Interpretable deconvolution of cell fractions via biologically-informed deep learning using scRNA-seq data
Source: PLoS Comput Biol. 2026 Aug 27;22(8):e1014727. doi: 10.1371/journal.pcbi.1014727 (PMC13552964; doi:10.1371/journal.pcbi.1014727)
Supplement: Supplementary file 7 [file pcbi.1014727.s007.pdf]

S1 Table. Summary of the single-cell RNA sequencing (scRNA-seq) reference dataset used in this study.

Tissue	Dataset	# cells	Data location	Original source
PBMC	PBMC8k	8381	`https://www.10xgenomics.com/datasets/8-k-pbm-cs-from-a-healthy-donor-2-standard-2-1-0`	10x Genomics (8k PBMCs from a Healthy Donor)
PBMC	PBMC6k	5400	`https://www.10xgenomics.com/datasets/6-k-pbm-cs-from-a-healthy-donor-1-standard-1-1-0`	10x Genomics (6k PBMCs from a Healthy Donor)
PBMC	DonorA	2900	`https://www.10xgenomics.com/datasets/frozen-pbm-cs-donor-a-1-standard-1-1-0`	10x Genomics (Frozen PBMCs (Donor A))
Brain	Song et al.	8467	`https://mai.fudan.edu.cn/stab/`	[36-39]
High-grade serous ovarian cancer (HGSC)	GSE232314	18392	`https://www.ncbi.nlm.nih.gov/geo/query/acc.cgi?acc=GSE232314`	[46]
Pancreatic islets	GSE84133	8569	`https://www.ncbi.nlm.nih.gov/geo/query/acc.cgi?acc=GSE84133`	[50]
Colorectal cancer (CRC)	E-MTAB-8410	52587	`https://www.ebi.ac.uk/biostudies/ArrayExpress/studies/E-MTAB-8410`	[53]
