# Supplementary material for: iDCF: Interpretable deconvolution of cell fractions via biologically-informed deep learning using scRNA-seq data
Source: PLoS Comput Biol. 2026 Aug 27;22(8):e1014727. doi: 10.1371/journal.pcbi.1014727 (PMC13552964; doi:10.1371/journal.pcbi.1014727)
Supplement: Supplementary file 8 [file pcbi.1014727.s008.pdf]

S2 Table. Relationship between CellTypist annotated cell types and major cell types.

**CellTypist**	**Major**
Classical monocytes<br>Non-classical monocytes	Monocyte
Tem/Effector helper T cells<br>Tcm/Naive helper T cells<br>Regulatory T cells	CD4 T cell
Tcm/Naive cytotoxic T cells<br>Tem/Temra cytotoxic T cells<br>Tem/Trm cytotoxic T cells<br>MAIT cells	CD8 T cell
Naive B cells<br>Memory B cells<br>B cells<br>Plasma cells	B cell
CD16+ NK cells<br>NK cells	NK cell
pDC<br>DC2<br>HSC/MPP	Unknown
