# Supplementary material for: iDCF: Interpretable deconvolution of cell fractions via biologically-informed deep learning using scRNA-seq data
Source: PLoS Comput Biol. 2026 Aug 27;22(8):e1014727. doi: 10.1371/journal.pcbi.1014727 (PMC13552964; doi:10.1371/journal.pcbi.1014727)
Supplement: Supplementary file 9 [file pcbi.1014727.s009.pdf]

S3 Table. Summary of the bulk RNA sequencing (RNA-seq) dataset used for deconvolution evaluation.

Tissue	Dataset	# samples	Experiment type	Data location	Original source
PBMC	Sdy67	12	RNA-seq	https://www.immport.org/shared/study/SDY67	[63]
PBMC	Monaco 1	12	RNA-seq	https://www.ncbi.nlm.nih.gov/geo/query/acc.cgi?acc=GSE107011	[64]
PBMC	GSE65133	20	Microarray	https://www.ncbi.nlm.nih.gov/geo/query/acc.cgi?acc=GSE65133	[65]
PBMC	GSE107572	9	RNA-seq	https://www.ncbi.nlm.nih.gov/geo/query/acc.cgi?acc=GSE107572	[66]
PBMC	GSE120502	250	RNA-seq	https://www.ncbi.nlm.nih.gov/geo/query/acc.cgi?acc=GSE120502	[67]
PBMC	Monaco 2	164	Microarray	https://www.ncbi.nlm.nih.gov/geo/query/acc.cgi?acc=GSE107990	[64]
PBMC	Sdy67_250	250	RNA-seq	https://www.immport.org/shared/study/SDY67	[63]
PBMC	GSE193141	156	RNA-seq	https://www.ncbi.nlm.nih.gov/geo/query/acc.cgi?acc=GSE193141	[68]
Brain	ROSMAP	41, 493*	RNA-seq	https://www.synapse.org/Synapse:syn3219045	[69, 13, 70]
HGSC	HGSC	41	RNA-seq	https://www.ncbi.nlm.nih.gov/geo/query/acc.cgi?acc=GSE232314	[47, 48]
Type-2 diabetes (T2D)	GSE50244	89**	RNA-seq	https://www.ncbi.nlm.nih.gov/geo/query/acc.cgi?acc=GSE50244	[49]
SARS-CoV-2	GSE159717	6	RNA-seq	https://www.ncbi.nlm.nih.gov/geo/query/acc.cgi?acc=GSE159717	[51]
SARS-CoV-2	GSE165890	12	RNA-seq	https://www.ncbi.nlm.nih.gov/geo/query/acc.cgi?acc=GSE165890	[52]
CRC-Small extracellular vesicles (CRC-sEVs)	TCGA-Colon adenocarcinoma	524	RNA-seq	https://portal.gdc.cancer.gov/	[55]

* 41 samples provided immunohistochemistry information, 493 samples provided Braak stage information.
** 77 samples provided hemoglobin A1c information.
