# Supplementary material for: iDCF: Interpretable deconvolution of cell fractions via biologically-informed deep learning using scRNA-seq data
Source: PLoS Comput Biol. 2026 Aug 27;22(8):e1014727. doi: 10.1371/journal.pcbi.1014727 (PMC13552964; doi:10.1371/journal.pcbi.1014727)
Supplement: Supplementary file 10 [file pcbi.1014727.s010.pdf]

S4 Table. Relationship between cell types in bulk datasets and major cell types.

Monaco 1	GSE65133	GSE107572	GSE120502	Monaco 2	Sdy67_250	GSE193141	Major
Monocytes C<br>Monocytes I<br>Monocytes NC	Monocytes	Monocytes	CD14	Monocytes	Monocytes C<br>Monocytes I<br>Monocytes NC	Monocytes	Monocyte
T CD4 Naïve<br>Tregs<br>Tfh<br>Th1<br>Th1/Th17<br>Th17<br>Th2<br>T CD4 TE	Naïve CD4 T cells<br>Resting memory CD4 T cells<br>Activated memory CD4 T cells	CD4+ T cells<br>Tregs	CD4	T CD4	T CD4	T cells CD4	CD4 T cell
T CD8 Naïve<br>T CD8 CM<br>T CD8 EM<br>T CD8 TE<br>MAIT	CD8 T cells	CD8+ T cells	CD8	T CD8	T CD8	T cells CD8	CD8 T cell
B Naïve<br>B NSM<br>B SM<br>B Ex<br>Plasmablasts	Naïve B cells<br>Memory B cells	B cells	CD20	B Memory<br>B Naïve<br>Plasmablasts	B Naïve<br>B Ex<br>B NSM<br>B SM<br>Plasmablasts	B cells	B cell
NK	NK cells	Natural killer cells	CD56	NK	NK	NK cells	NK cell
pDCs<br>mDCs<br>Progenitors<br>T gd Vd2<br>T gd non-Vd2<br>Neutrophils LD<br>Basophils LD	Gamma delta T cells	Myeloid dendritic cells<br>Neutrophils	Unknown*	Unknown*	pDCs<br>mDCs<br>Basophils LD<br>Unknown	Unknown**	Unknown

* The proportion of "Unknown" for GSE120502 and Monaco 2 was calculated as 1 minus the sum of the proportions of all other specified cell types.

** The proportion of "Unknown" for GSE193141 was calculated as 1 minus the sum of the proportions of Granulocytes, Lymphocytes, and Monocytes.
