# Supplementary material for: iDCF: Interpretable deconvolution of cell fractions via biologically-informed deep learning using scRNA-seq data
Source: PLoS Comput Biol. 2026 Aug 27;22(8):e1014727. doi: 10.1371/journal.pcbi.1014727 (PMC13552964; doi:10.1371/journal.pcbi.1014727)
Supplement: Supplementary file 11 [file pcbi.1014727.s011.pdf]

**S5 Table. Performance comparison (Pearson correlation coefficient $r$) of different deconvolution methods across PBMC6k and DonorA datasets.**

| Method | B cell | CD4 T cell | CD8 T cell | Monocyte | NK cell | $r$ median |
|---|---|---|---|---|---|---|
| **Dataset: PBMC6k** | | | | | | |
| iDCF | 0.9980 | 0.9830 | 0.8816 | 0.9981 | 0.9914 | **0.9914** |
| DeSide | 0.9939 | 0.8967 | 0.7723 | 0.9923 | 0.9709 | 0.9709 |
| scpDeconv | 0.8466 | 0.7131 | 0.3572 | 0.7004 | 0.8253 | 0.7131 |
| TAPE-O | 0.9896 | 0.7766 | 0.8129 | 0.9920 | 0.9706 | 0.9706 |
| TAPE-H | 0.9909 | 0.7154 | 0.8038 | 0.9921 | 0.9388 | 0.9388 |
| Scaden | 0.9969 | 0.9444 | 0.8229 | 0.9973 | 0.9850 | 0.9850 |
| ReDeconv | 0.9970 | 0.9736 | 0.9390 | 0.9967 | 0.9866 | 0.9866 |
| BayesPrism | 0.9947 | 0.9752 | 0.9643 | 0.9960 | 0.9906 | 0.9906 |
| CIBERSORTx | 0.9968 | 0.9595 | 0.8271 | 0.9972 | 0.9814 | 0.9814 |
| **Dataset: DonorA** | | | | | | |
| iDCF | 0.9975 | 0.9745 | 0.7938 | 0.9980 | 0.9933 | **0.9933** |
| DeSide | 0.9917 | 0.8931 | 0.6622 | 0.9930 | 0.9774 | 0.9774 |
| scpDeconv | 0.8429 | 0.7927 | 0.3634 | 0.7563 | 0.8706 | 0.7927 |
| TAPE-O | 0.9946 | 0.9688 | 0.7807 | 0.9960 | 0.9898 | 0.9898 |
| TAPE-H | 0.9949 | 0.9644 | 0.8089 | 0.9951 | 0.9835 | 0.9835 |
| Scaden | 0.9969 | 0.9656 | 0.7237 | 0.9975 | 0.9902 | 0.9902 |
| ReDeconv | 0.9944 | 0.9478 | 0.8548 | 0.9958 | 0.9857 | 0.9857 |
| BayesPrism | 0.9941 | 0.9595 | 0.9049 | 0.9948 | 0.9931 | 0.9931 |
| CIBERSORTx | 0.9975 | 0.9488 | 0.6876 | 0.9971 | 0.9916 | 0.9916 |
