# Supplementary material for: iDCF: Interpretable deconvolution of cell fractions via biologically-informed deep learning using scRNA-seq data
Source: PLoS Comput Biol. 2026 Aug 27;22(8):e1014727. doi: 10.1371/journal.pcbi.1014727 (PMC13552964; doi:10.1371/journal.pcbi.1014727)
Supplement: Supplementary file 12 [file pcbi.1014727.s012.pdf]

S6 Table. Evaluation of deconvolution performance based on CCC and MAE across different cell types.

Method	B cell		CD4 T cell		CD8 T cell		Monocyte		NK cell		Unknown	
	CCC	MAE	CCC	MAE	CCC	MAE	CCC	MAE	CCC	MAE	CCC	MAE
iDCF	**0.5810**	**0.0224**	**0.5203**	**0.0893**	**0.5278**	**0.0702**	0.2825	0.1786	0.3617	0.0545	0.0471	**0.0489**
DeSide	0.3631	0.0295	0.2734	0.1174	0.3505	0.0759	0.1902	0.1790	0.2747	0.0490	0.0451	0.0528
scpDeconv	0.2257	0.0875	0.2221	0.1605	0.1078	0.1161	0.2881	0.1217	0.1562	0.1384	0.0090	0.1151
TAPE-O	0.2400	0.0540	0.2549	0.1052	0.2089	0.0912	0.3838	0.0911	0.1693	0.0652	0.0028	0.0683
TAPE-H	0.2936	0.0395	0.2009	0.1147	0.2422	0.0940	0.3960	0.0879	0.1474	0.0687	0.0067	0.0827
Scaden	0.3824	0.0322	0.4186	0.1046	0.3487	0.0947	0.2315	0.1804	0.2676	0.0699	0.0110	0.0601
ReDeconv	0.5253	0.0285	0.3296	0.1315	0.2996	0.1052	0.0855	0.3013	**0.4025**	**0.0478**	-0.0967	0.0753
BayesPrism	0.4243	0.0429	0.1566	0.2184	0.2407	0.1169	0.3975	0.0929	0.3096	0.0722	0.0390	0.1182
CIBERSORTx	0.2466	0.0615	0.4956	0.0924	0.2948	0.1100	**0.4702**	**0.0901**	0.2332	0.0776	**0.0539**	0.0544

Note: Higher CCC values and lower MAE values indicate better deconvolution performance. Bold text highlights the best-performing method for each cell type.
