# Supplementary material for: iDCF: Interpretable deconvolution of cell fractions via biologically-informed deep learning using scRNA-seq data
Source: PLoS Comput Biol. 2026 Aug 27;22(8):e1014727. doi: 10.1371/journal.pcbi.1014727 (PMC13552964; doi:10.1371/journal.pcbi.1014727)
Supplement: Supplementary file 13 [file pcbi.1014727.s013.pdf]

S7 Table. Statistical comparison of deconvolution performance between Control and SARS-CoV-2 groups across different methods.

**Method**	**Control** (Mean ± SEM)	**SARS-CoV-2** (Mean ± SEM)	*p*-**value**
iDCF	0.5086 ± 0.0028	0.4743 ± 0.0047	1.0642×10^{-4}
DeSide	0.5547 ± 0.0033	0.5272 ± 0.0008	1.3550×10^{-4}
scpDeconv	0.0451 ± 0.0027	0.0419 ± 0.0033	0.2305
TAPE-O	0.5887 ± 0.0014	0.5603 ± 0.0037	1.3415×10^{-4}
TAPE-H	0.5517 ± 0.0019	0.5232 ± 0.0044	3.1829×10^{-4}
Scaden	0.5680 ± 0.0059	0.5510 ± 0.0050	0.0262
ReDeconv	0.2905 ± 0.0043	0.2633 ± 0.0059	0.0024
BayesPrism	0.1714 ± 0.0041	0.1554 ± 0.0061	0.0284
CIBERSORTx	0.1988 ± 0.0036	0.1971 ± 0.0018	0.3379

Note: *p*-values are determined by one-sided hypothesis testing. SEM stands for standard error of the mean.
